# An Update on Nondopaminergic Treatments for Motor and Non-motor Symptoms of Parkinson’s Disease

**DOI:** 10.2174/1570159X20666220222150811

**Published:** 2023-06-15

**Authors:** Xiao-Zhong Jing, Xiang-Zhen Yuan, Xingguang Luo, Shu-Yun Zhang, Xiao-Ping Wang

**Affiliations:** 1 Department of Neurology, TongRen Hospital, Shanghai Jiao Tong University School of Medicine, Shanghai, China;; 2 Department of Neurology, Weifang People's Hospital, Weifang, Shandong, China;; 3 Department of Psychiatry, Yale University School of Medicine, New Haven, CT, USA

**Keywords:** Parkinson’s disease, nondopaminergic treatments, nondopaminergic neurotransmitters, motor symptoms, non-motor symptoms, update

## Abstract

Nondopaminergic neurotransmitters such as adenosine, norepinephrine, serotonin, glutamate, and acetylcholine are all involved in Parkinson's disease (PD) and promote its symptoms. Therefore, nondopaminergic receptors are key targets for developing novel preparations for the management of motor and non-motor symptoms in PD, without the potential adverse events of dopamine replacement therapy. We reviewed English-written articles and ongoing clinical trials of nondopaminergic treatments for PD patients till 2014 to summarize the recent findings on nondopaminergic preparations for the treatment of PD patients. The most promising research area of nondopaminergic targets is to reduce motor complications caused by traditional dopamine replacement therapy, including motor fluctuations and levodopa-induced dyskinesia. Istradefylline, Safinamide, and Zonisamide were licensed for the management of motor fluctuations in PD patients, while novel serotonergic and glutamatergic agents to improve motor fluctuations are still under research. Sustained-release agents of Amantadine were approved for treating levodopa induced dyskinesia (LID), and serotonin 5HT_1B_ receptor agonist also showed clinical benefits to LID. Nondopaminergic targets were also being explored for the treatment of non-motor symptoms of PD. Pimavanserin was approved globally for the management of hallucinations and delusions related to PD psychosis. Istradefylline revealed beneficial effect on daytime sleepiness, apathy, depression, and lower urinary tract symptoms in PD subjects. Droxidopa may benefit orthostatic hypotension in PD patients. Safinamide and Zonisamide also showed clinical efficacy on certain non-motor symptoms of PD patients. Nondopaminergic drugs are not expected to replace dopaminergic strategies, but further development of these drugs may lead to new approaches with positive clinical implications.

## 1 INTRODUCTION

Parkinson's disease (PD) is a multi-system disease characterized by bradykinesia, resting tremor, rigidity, abnormal gait and posture, as well as non-motor symptoms including olfactory disorder, sleep disorder, autonomic nerve dysfunction, cognitive and mental disorder [1]. Dopaminergic neurotransmitter system has always been the main target of pharmacological therapies for PD symptoms since the discovery of striatal dopamine degeneration in the substantia striatal system of PD patients [2]. Dopamine replacement with levodopa is still the most efficient therapy for the management of primary features of PD. However, certain features may become an insufficient response to levodopa and chronic levodopa treatment may lead to motor complications. Due to the fluctuation of motor behavior and the assumptions of levodopa neurotoxicity, there has been a debate about the use of levodopa in the scientific community for many years [3, 4]. How to prevent and relieve motor fluctuations and levodopa-induced dyskinesia (LID) have become an important factor to be considered in PD treatment. In addition, adverse effects of dopamine receptor agonist (DA) include withdrawal syndrome, excessive daily somnolence as well as impulse control disorders, which considerably limit their clinical applications [5-7].

**Fig. (1):**
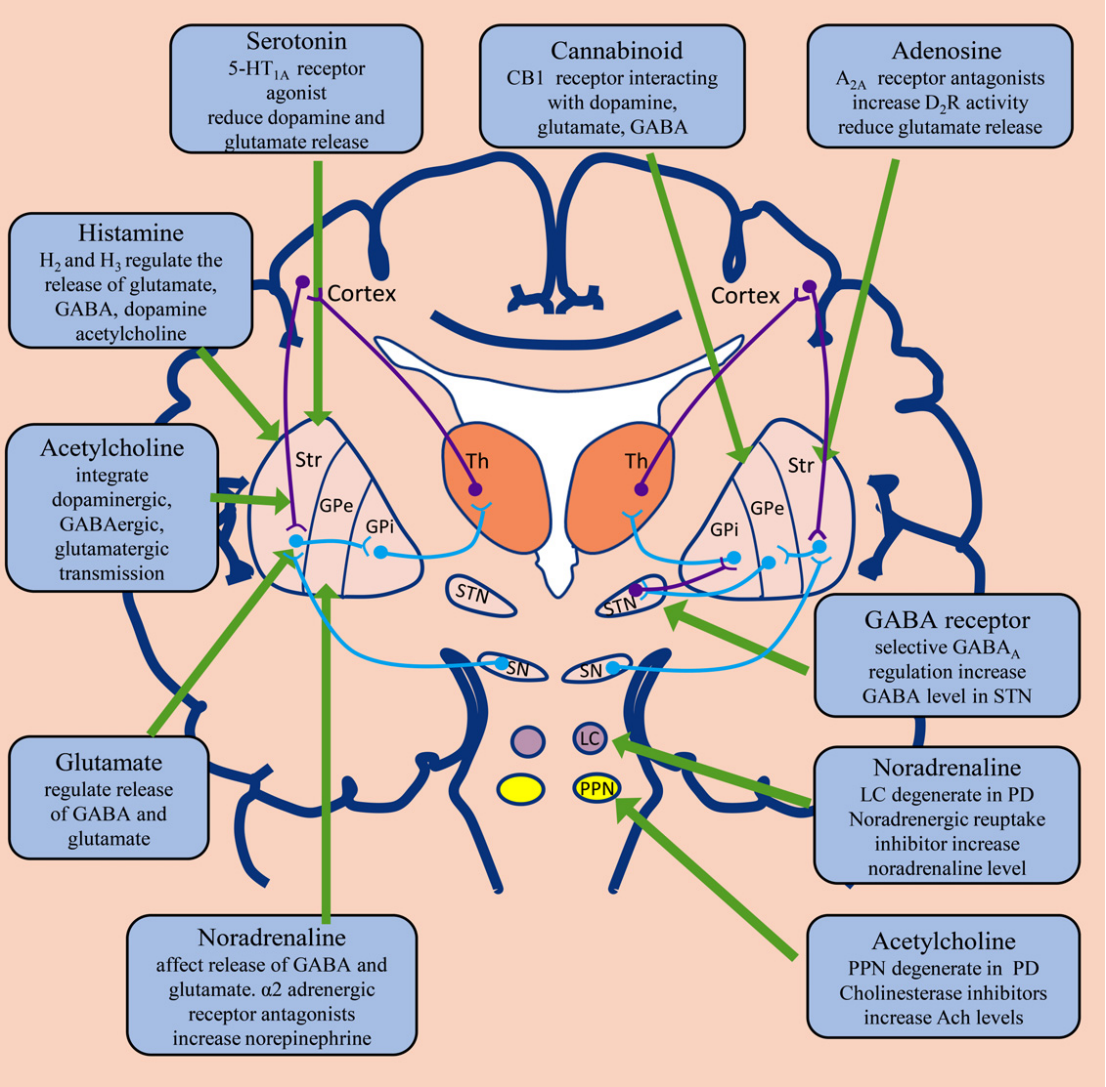
Nondopaminergic neurotransmitters including GABA, Glutamate, Acetylcholine, Histamine, Serotonin and Adenosine are distributed throughout the basal ganglia or related structures and play an important role in the motor and non-motor symptoms of PD patients. Nondopaminergic agents targeting on Histamine, Glutamate, Acetylcholine, Serotonin or Noradrenaline systems appear to regulate the direct pathway through the globus pallidus internus (**left**). While those targeting on GABA and adenosine systems mainly influence the indirect pathway reaching the globus pallidus externus and subthalamic nucleus before the globus pallidus internus (**right**). GPi: globus pallidus internus. SN: substantia nigra. PPN: pedunculopontine nucleus. GPe: globus pallidus externus. LC: locus coeruleus. Th: thalamus. STN: subthalamic nucleus. Str: striatum.

**Fig. (2):**
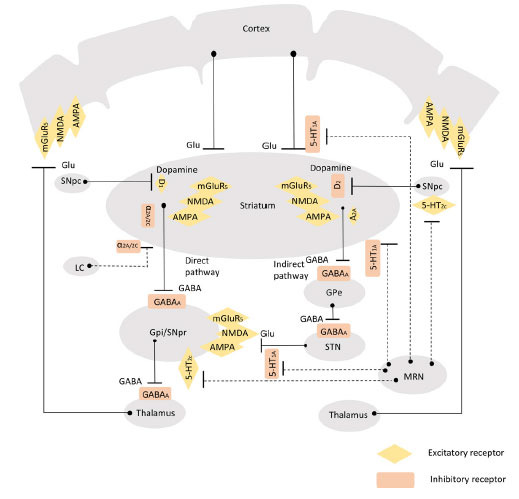
Schematic diagram of direct and indirect pathway in basal ganglia, regulation of serotonergic and noradrenergic projections, and receptors associated with non-dopaminergic treatments. GPi: globus pallidus internus. SNpc: substantia nigra pars compacta. pedunculopontine nucleus. GPe: globus pallidus externus. STN: subthalamic nucleus. LC: locus coeruleus. SNpr substantia nigra pars reticulata. MRN: median raphe nucleus.

Nondopaminergic neurotransmitters such as serotonin, adenosine, glutamate, norepinephrine, as well as acetylcholine are all considered to be involved in the regulation of basal ganglia and other neural circuits important for movement [8-10] (Figs. **1** and **2**). The concept of targeting nondopaminergic receptors has been a key point of study for years as a possible way to alleviate motor and non-motor symptoms of PD, without the potential adverse events of dopamine replacement. Several studies have reported that novel anti-PD agents targeting α2-adrenergic receptors, adenosine A2A receptors, glutamate receptors and monoamine oxidase-B could improve clinical efficacy, prevent the occurrence of motor complications as well as alleviate LID [11-15]. Different studies have also evaluated nondopaminergic preparations as potential therapeutic options for non-motor symptoms of PD sufferers. Research and development of novel agents that act on nondopaminergic neurotransmitters may be a new direction for the treatment of PD in the future.

Therefore, this review aims to provide a comprehensive summary of the emerging findings on nondopaminergic therapy and the role of each compound in the control of motor complications and non-motor symptoms. A comprehensive knowledge of recent literature evidence would help clinicians in the management of nondopaminergic therapy for PD patients.

## 2 SEARCH STRATEGY AND SELECTION CRITERIA

**Fig. (3):**
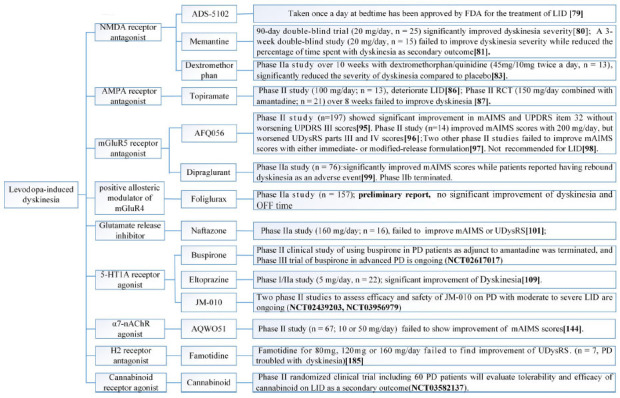
Nondopaminergic preparations for the management of Levodopa-induced dyskenia in PD patients.

**Fig. (4):**
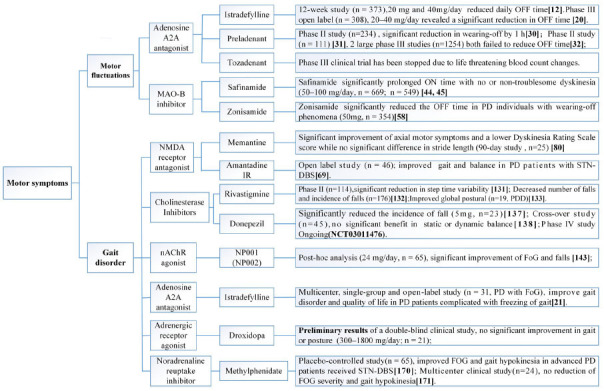
Nondopaminergic preparations for the management of motor fluctuations and gait disorder.

**Fig. (5):**
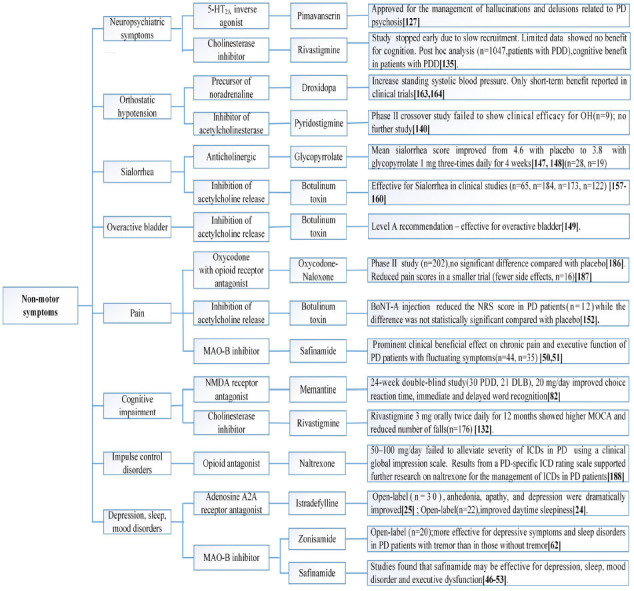
Nondopaminergic preparations for the management of non-motor symptoms in PD patients.

We reviewed English-written articles published in PubMed between January 2014 and November 2021 by using the following key terms ‘Parkinson's disease’, ‘nondopaminergic treatments’, adenosine’, ‘acetylcholine’, ‘histamine’, ‘glutamate’, ‘serotonin’, ‘5HT’, ‘noradrenaline’, ‘γ-aminobutyric acid’, ‘GABA’, ‘opioid’, ‘cannabinoid’, ‘motor symptoms’, ‘motor fluctuations’, ‘LID’, ‘dyskinesia’ and ‘non-motor symptoms’. Ongoing clinical trials were also searched using similar key terms on https://clinicaltrials.gov. In most cases, papers were only selected for review when there was an established rating scale or well-described measurement of endpoints. Systematic reviews and meta-analysis were included if they reported nondopaminergic treatment for PD. Studies with insufficient data, duplicated publications, and drugs being tested on animals were excluded. Important nondopaminergic targets that were previously evaluated were also mentioned if no further studies had been performed using this target since 2014. The text structure is organized based on the different nondopaminergic targets. Readers are referred to (Figs. **3-5**) for categorization of nondopaminergic targets based on symptoms. A comprehensive review of the preclinical background and scientific principles for the use of nondopaminergic targets are beyond the scope of this manuscript and readers may refer to referenced related reviews in each section.

## 3 ADENOSINE A2A RECEPTOR ANTAGONISTS

Antagonists of adenosine A2A receptors have been reported to moderately improve clinical symptoms in advanced PD patients without any impairment to motor symptoms [16]. Adenosine A2A receptor is strongly expressed in the striatum and can interact with a variety of receptors. Adenosine A2A receptor antagonists can promote the dopamine D2 receptor signaling pathway and show certain anti-Parkinson activity (Figs. **1** and **2**) [17]. Interaction with metabotropic glutamate receptor subtype 5 (mGluR5) can reduce the release of excitatory neurotransmitter glutamate or the phosphorylation of glutamate receptors, thereby alleviating neurotoxicity [17]. Adenosine A2A receptor antagonists are some of the most promising compounds for the management of PD by reversing exercise injury and contrasting cell degeneration. Currently, new antiparkinsonian agents targeting adenosine A2A receptors are in clinical trials.

Istradefylline is a selective adenosine A2A receptor antagonist developed by Kyowa Hakko Kogyo Company in Japan. A pooled analysis of 723 subjects found that Istradefylline was beneficial to motor symptoms of PD sufferers by activating the GABA pathway in the subthalamic nucleus [18]. Two randomized clinical studies with a total of 681 PD patients showed that Istradefylline reduced OFF time and increased ON time without dyskinesia compared to placebo [19, 20]. A multicenter, single-group and prospective study including 31 PD patients found that Istradefylline could ameliorate gait disorder and quality of life in PD sufferers complicated with freezing of gait (FOG) [21]. A pooled analysis of 723 PD individuals revealed that in patients over 65 years, the use of Istradefylline provided a significant beneficial response by reducing OFF time and increasing ON time. OFF time reduction was more favorable in patients with more than 8-hour daily OFF time at baseline. The improvement in Unified Parkinson’s Disease Rating Scale (UPDRS) Part III score was beneficial in PD subjects with UPDRS Part III baseline score over 20 [22].

Animal experiments reported that adenosine A2A receptor antagonist could inhibit bladder overactivity in PD mouse model. In 2016, a clinical study of 13 male PD subjects found that Istradefylline could improve the lower urinary tract symptoms and relieve urination and storage symptoms in PD patients [23]. A 3-month clinical trial including 22 PD patients explored the therapeutic effect of Istradefylline on sleep disorders, daytime drowsiness, and motor symptoms. The results revealed that Istradefylline could significantly improve daytime sleepiness in PD patients, which may be due to its effect of increasing alertness, while it has no effect on sleep disorders [24]. Another open-label trial of 30 PD subjects to access the effect of Istradefylline on mood disorders in PD patients found significant improvement in anhedonia, apathy, and depression in the Istradefylline treatment group [25]. Recently, a sub-analysis of a 1-year observational study including 732 PD patients was performed to evaluate the effect of Istradefylline on non-motor symptoms in Istradefylline-naïve Japanese PD individuals. The study found no significant improvement in UPDRS part 1 total score or any sub-items, or in the PD questionnaire (PDQ-8) total score at the end of the 52-week observational period [26]. A pilot investigation of 126 inpatients with PD showed benefit for PD patients following an inpatient setting in a specialized PD unit. The study found that the severity of non-motor symptoms also depends on the severity of motor symptoms [27]. Therefore, the improvement of non-motor symptoms in PD patients may relate to the improvement of motor symptoms. Further research is still required to investigate the precise effect of Istradefylline on non-motor symptoms in PD patients. Istradefylline was approved as a combined treatment for levodopa in Japan in 2013.

Preladenant is another potent and selective A2A antagonist which has been found to improve motor function in animal models of PD [28, 29]. A phase II and dose-finding clinical trial of 234 PD subjects found that 5mg and 10mg preladenant twice a day had a certain clinical significance in reducing OFF time in PD individuals receiving levodopa or other antiparkinsonian agents [30]. However, subsequent studies did not confirm the effectiveness of reducing OFF periods. Another phase II clinical study on 111 Japanese PD patients receiving a stable dose of levodopa showed that Preladenant failed to reduce mean OFF time from baseline to week 12. The placebo effect, disadvantages of using paper diaries as well as the possible confounding factor of caffeine are all alleged reasons behind this negative test [31]. In two phase III adjunctive studies of 1254 PD patients from multicenter, Preladenant as an adjunct therapy to levodopa did not find significant improvement in OFF time compared to placebo. However, no statistically significant treatment difference was found in the active comparator Rasagiline treatment group, thus making the authors speculate that the study failed and was insufficient to evaluate the potential effect of Preladenant for the treatment of PD [32]. An international study with a total of 1,007 PD subjects to explore the effectiveness and tolerability of a series of Preladenant doses as monotherapy for newly diagnosed PD individuals who were not treated with levodopa or DA also failed to support the effectiveness of Preladenant [33]. Preladenant was safe and had a good tolerance, with reported side effects including constipation, nausea, somnolence and worsening of PD [31, 32]. Owing to the negative results of the above clinical trials, no further research was conducted on Preladenant for the treatment of PD.

Tozadenant is a candidate agent for selective adenosine A2A receptor antagonist. A large-scale clinical trial enrolled 420 PD patients taking levodopa for more than 12 months and suffering wearing-off motor fluctuations with an OFF period lasting for more than 2.5 h a day [34]. The results indicated that Tozadenant 120 mg or 180 mg twice daily was safe and had a promising therapeutic effect for reducing the daily OFF period [34]. The common adverse reactions of Tozadenant were dyskinesia, nausea, dizziness, and constipation. Deterioration of PD symptoms has also been reported, which should be monitored carefully. Tozadenant is now in phase III clinical trial. Unfortunately, the program was stopped due to life-threatening blood count changes.

Studies consistently linked the use of Caffeine, a nonselective antagonist of adenosine receptors, to a lower risk of PD, although the mechanism behind this robust finding is unclear [35]. Recent studies have evaluated the effect of Caffeine on the symptoms of PD. A 6-week open-label study of 48 PD individuals found that the maximum tolerance of Caffeine in PD patients appears to be 200 mg to 400 mg daily, and caffeine for 400 mg/day showed potential beneficial effects on motor function and excessive daytime sleepiness [36]. A small clinical trial of Caffeine in 61 PD patients with daytime sleepiness found that Caffeine up to 200 mg twice a day for 26 weeks did not significantly improve excessive daytime somnolence in PD patients. The study observed improvements in total UPDRS score and the objective motor component in Caffeine treatment group compared with placebo [37]. However, a multicenter parallel-group controlled trial involving 60 PD patients who received Caffeine and 61 subjects who received placebo showed that caffeine 400 mg per day did not significantly improve motor manifestations of PD. The epidemiological link between Caffeine and a lower risk of PD did not seem to be explained by symptomatic effect [38]. No side effects were more common in Caffeine compared with placebo. Whether Caffeine could improve the motor symptoms of PD still needs further research.

As a class of drugs, the advantage of A2A receptor antagonists over monoamine oxidase B (MAO-B) inhibitor and catechol-O-methyltransferase (COMT) inhibitor is that they do not interact with narcotics, tyramine, and antidepressants. Based on successful preclinical and/or phase I studies, there are other A2A receptor antagonists being considered for the expected phase II study of PD, such as ST4206, V81444, PBF-509 and ST1535.

## 4 MONOAMINE OXIDASE-B INHIBITORS

Brain function depends largely on the neurotransmission of biogenic monoamines. Their metabolism utilizes MAO-B in neuronal and glial cells and thus, inhibition of MAO-B increases the biogenic amine levels [39]. Inhibiting the activity of MAO-B increases the concentration of dopamine in the brain and protect neurons. MAO-B inhibitors can be used alone or in combination with levodopa to delay the occurrence of motor complications and reduce levodopa dosage in PD patients [40, 41]. Safamide and Zonisamide are clinically available reversible MAO-B inhibitors that increase dopamine levels *via* reducing levodopa decomposition. They also inhibit glutamate release by blocking voltage-gated sodium and calcium channels.

Safinamide is a highly selective and reversible MAO-B inhibitor developed by Newron. It can block N-type calcium channels and voltage-dependent sodium channel as well as inhibit the release of excitatory neurotransmitter glutamate [42]. Safinamide 100 mg or 200mg/day showed good tolerability as adjunctive therapy for early PD patients receiving DA therapy [43]. Borgohain *et al.* conducted a 24-week multicenter clinical study including 669 PD individuals to explore the effectiveness and safety of Safinamide (50, 100 mg/d) in the treatment of advanced PD patients with dyskinesia [44]. The study found that the ON time was prolonged by 1.37h in 50 mg/d group, 1.36h in 100 mg/d group and 0.97h in placebo group. In addition, Safinamide significantly improved OFF time, clinical status, and motor function in each group compared with placebo, and higher doses also improved activities of daily living and quality of life [44]. Another clinical study using Safinamide as an adjunctive therapy for 549 patients with moderate-to-advanced PD accompanied by motor fluctuations showed improvement of dyskinesia in patients who suffered moderate dyskinesia at the baseline level. This clinical trial also found that Safinamide had a significant clinical beneficial effect on OFF-time, ON-time, motor symptoms, quality of life and depressive symptoms [45].

The clinical efficacy of Safinamide for non-motor symptoms in PD subjects was also evaluated. Two Post hoc analyses with a total of 1021 PD patients found that Safinamide 100mg significantly improved the Parkinson’s Disease Questionnaire (PDQ-39) “Emotional well-being” domain scores, GRID Hamilton Rating Scale for Depression and health-related quality of life [46, 47]. In 2018, a post hoc analysis of 669 PD subjects assessed the long-term efficacy of Safinamide on chronic pain in fluctuating PD patients. According to the study, a significantly higher proportion of patients in the Safinamide group did not receive analgesic treatment compared to those in the placebo group (76.1% *versus* 70%). After 2 years of treatment, the proportion of patients in the Safinamide group who were not taking the painkiller increased significantly [48]. The secondary analysis of an open-label study [49] of 44 PD patients also reported that Safinamide was well tolerated and alleviated pain in PD patients at 6 months [50]. In 2020, an exploratory study involving 35 PD patients found that the addition of Safinamide ameliorated executive dysfunction in PD patients with symptom fluctuations at the end of levodopa dose. Specifically, the addition of Safinamide significantly improved attention and inhibited cognitive interference [51]. A multicenter retrospective study including 82 PD patients found significant improvement on the Hamilton Depression Scale after taking safinamide for 3 months, suggesting that Safinamide may be an efficacious treatment for depression in PD patients [52]. An open-label prospective study of 50 PD individuals found that Safinamide was well tolerated and improved non-motor symptoms of PD patients, including mood, sleep, daytime somnolence, cognition, gastrointestinal as well as urinary symptoms at 6 months [49]. Secondary analysis of the study also found a significant decrease in Non-Motor Symptoms Scale mood/apathy domain, and PDQ-39 emotional well-being domain [53]. Patients taking Safinamide were more likely to report adverse events, including dyskinesia, insomnia, dizziness, headache, nausea and falls [15, 43, 44]. The safety and efficacy of Safinamide on non-motor symptoms should be confirmed in larger randomized controlled trials. On February 26, 2015, Safinamide was approved for the first time in the European Union as a supplement to a stable dose of levodopa (used alone or combined with other anti-PD agents) for the treatment of advanced PD patients with motor fluctuations [54].

Zonisamide, a clinically available reversible MAO-B inhibitor, was introduced as an antiepileptic agent in Japan in 1989. *In vivo* evidence suggested that Zonisamide also reduced glutamate release. A phase II clinical trial found that both 25 mg/day and 50 mg/day of Zonisamide improved UPDRS Part III score and were well tolerated [55]. A small open-label study of 11 PD patients showed that Zonisamide 25 mg for 28 weeks was safe and efficient for improving motor functions in early-stage PD patients who were already receiving small amounts of levodopa [56]. A multicenter, parallel-treatment study including 185 advanced PD patients reported that Zonisamide 25 mg/day and 50 mg/day were efficacious and well tolerated for advanced PD [57]. Another clinical trial of 354 PD patients found that 50 mg of Zonisamide significantly decreased the OFF time in PD individuals with wearing-off phenomena [58]. Recently, a phase II study involving 137 subjects discovered that Zonisamide 50 mg as an adjunctive treatment with levodopa for 12 weeks significantly improved the UPDRS part III score in patients with dementia with Lewy bodies (DLB) [59]. Another phase III study of 346 subjects and a post hoc analysis of two randomized clinical studies also reported that Zonisamide improved parkinsonism in DLB patients and with good tolerability [60, 61]. The results from an open label trial of 20 PD patients with motor fluctuation also demonstrated the beneficial effect of Zonisamide 25 and 50 mg/day on motor symptoms and sleep disturbances in PD patients with motor fluctuation [62]. Zonisamide may be more efficacious for sleep disorders and depressive symptoms in PD patients troubled with tremor than in those without tremor [62]. In 2009, Zonisamide 25 mg/day was approved in Japan as adjunctive therapy for PD patients with insufficient response to levodopa treatment.

## 5 GLUTAMATERGIC PATHWAYS

### 5.1 Glutamate Receptor Antagonist

Glutamate is a crucial excitatory neurotransmitter in the brain and mediates its action by acting on ionotropic (N-methyl-D-aspartate [NMDA]) receptors, α-amino-3-hydroxy-5-methyl-4-isoxazole propionic (AMPA) and metabotropic glutamate receptors (mGluR1-mGluR8) (Figs. **1** and **2**) [63]. Inhibition of glutamate release can effectively relieve symptoms of PD [64, 65]. NMDA receptors belong to ligand-gated subtype of ionotropic glutamate receptors and are strongly expressed in dopaminergic neurons of substantia nigra, while mGluRs are predominantly expressed in the basal ganglia [66]. NMDA receptors play an essential part in regulating glutamatergic synaptic transmission and have a remarkable curative effect on LID.

Amantadine immediate release (IR) is a low affinity and noncompetitive glutamate receptor antagonist, which is efficacious and clinically useful for the treatment of LID [67, 68]. A study of 46 PD subjects who received subthalamic nucleus deep brain stimulation(STN-DBS)found that Amantadine IR has clinical benefits on gait and balance in PD individuals who received STN-DBS [69]. Data from a phase IV washout clinical study of 57 amantadine-treated dyskinetic patients with PD emphasize the fact that Amantadine IR can be effective for LID in the long term while withdrawing of Amantadine significantly aggravated LID wihtin a mean time of 7 days. Therefore, clinicians should be cautious about the discontinuing of Amantadine due to the significant and uncomfortable worsening of LID [70].

ADS-5102 is a long-acting and sustained-release Amantadine hydrochloride capsule formulation. Amantadine alleviates parkinsonian symptoms by increasing extracellular dopamine levels in the striatum through reuptake inhibition and NMDA antagonism [71]. In 2015, a randomized phase II/III study of 83 subjects investigated the safety, effectiveness as well as tolerability of ADS-5102 in the management of LID [72]. Patients received three different doses (260 mg, 340 mg, and 420 mg) of ADS-5102 (amantadine hydrochloride) or placebo once daily for 8 weeks. The results showed that ADS-5102 340 mg per day at bedtime was well tolerated and efficacious for relieving dyskinesia in PD patients. In addition, ADS-5102 dramatically increased ON time without causing dyskinesia according to patient diaries [72]. In 2017, a phase III clinical trial involving 77 patients evaluated the effectiveness and safety of 274 mg ADS-5102 sustained release capsule (equivalent to 340 mg Amantadine hydrochloride) in the management of LID [73]. Results from the study showed that ADS-5102 274 mg significantly reduced LID and ameliorated OFF time. Another clinical study involving 126 patients confirmed the value of ADS-5102 274 mg taken orally at bedtime in relieving or eliminating LID and OFF time [74]. Post hoc analysis of 196 PD patients from two randomized clinical trials found that ADS-5102 was efficacious for depression and daytime sleepiness in PD patients troubled with dyskinesia and the improvement of non-motor symptoms was related to the improvement of dyskinesia [75].

ADS-5102, as a sustained-release Amantadine preparation, provides a more continuous and higher plasma concentration of Amantadine than immediate-release preparations [76, 77]. ADS-5102 only needs to be administered once daily at bedtime, which simplifies the sometimes rather complex drug intake regimen, especially for patients with advanced PD who have well-known compliance issues [76, 78]. The application of ADS-5102 is more convenient and simpler than deep brain stimulation or an infusion regimen [76]. Reported adverse effects included constipation, nausea, dizziness, and dry mouth [14, 73, 74, 76]. ADS-5102 for 274 mg, taken once a day at bedtime, has been approved by FDA for the treatment of LID [79].

Memantine is another non-selective NMDA receptor antagonist primarily used for the management of Alzheimer's disease (AD). The anti-dyskinetic effects of Memantine on PD patients have been studied before, while the results have not provided convincing evidence for the treatment of LID. In a small clinical trial of 25 PD patients, Memantine 20 mg/day for 90 days was associated with improved axial motor symptoms and lower Dyskinesia Rating Scale score [80]. Another small clinical trial of 15 PD subjects observed that Memantine 20 mg/day for 3 weeks significantly reduced the daytime duration of dyskinesia in PD patients, although the study failed to reach the primary endpoint of dyskinesia severity [81]. These two small clinical trials indicated that Memantine may have therapeutic effects on dyskinesia in PD subjects and a larger clinical study with a longer observation time is warranted. A double-blind clinical trial of 51 subjects (30 with Parkinson's disease dementia (PDD) and 21 with dementia with Lewy bodies (DLB) found that Memantine at 20 mg improved choice reaction time, immediate and delayed word recognition in both PDD and DLB groups [82]. So far, there has been no further study of Memantine in the treatment of PD.

Dextromethorphan (combined with a low dose of CYP2D6 inhibitor quinidine to increase bioavailability) has been licensed for the administration of pseudobulbar affect. Dextromethorphan has been studied for the treatment of LID owing to its major NMDA antagonist activity and effect on serotonin and norepinephrine reuptake inhibitors. A phase IIa clinical study of 13 PD patients found that Dextromethorphan/quinidine 45 mg/10 mg twice a day for 2 weeks significantly reduced the severity of dyskinesia compared with placebo [83]. A larger clinical trial is needed to confirm this conclusion, while no further research is currently planned.

### 5.2 AMPA Receptor Antagonists

Topiramate is a commonly used antiepileptic with a variety of mechanisms, such as inhibition of glutamatergic activity *via* AMPA and kainic acid receptors. Preclinical studies showed that Topiramate significantly reduced LID and may synergize with Amantadine [84, 85]. However, clinical trials did not find that topiramate could improve LID. A clinical study involving 13 PD patients showed that topiramate 100 mg/day for 4 weeks worsened LID and was poorly tolerated by PD patients [86]. Another clinical trial including 21 PD patients also found that topiramate 25-150 mg/day in combination with Amantadine over 8 weeks failed to improve dyskinesia [87]. Reported adverse events included worsening dyskinesia, hallucinations, dry eyes/mouth and breathing problems. Other AMPA antagonists such as Perampanel and Remacemide have been discussed in previous studies for the lack of efficacy and poor tolerance in relieving motor symptoms and LID in PD sufferers [88, 89]. AMPA receptor antagonists do not seem to be very promising in the treatment of PD based on the results of previous studies.

### 5.3 mGluR4 Receptor Positive Allosteric Modulators

mGluR4 negatively regulates the release of γ-aminobutyric acid (GABA) and glutamate in the indirect pathway of basal ganglia and is therefore considered to be a potential target for the treatment of motor symptoms in PD patients [90]. Animal study found that Foliglurax, a positive allosteric modulator of mGluR4 could ameliorate motor symptoms and motor complications in macaque models of PD [91]. A 28-day phase IIa study involving 157 PD individuals with motor fluctuations was conducted to explore the efficacy of Foliglurax. A recent press release reported that there had been no significant improvement of dyskinesia and OFF time, and no tolerance problems were found. Full details of the phase IIa study are still pending, and no further development of such agents has been reported.

### 5.4 mGluR5 Receptor Antagonists

In preclinical studies, increased specific binding of mGluR5 in the posterior putamen and globus pallidus of primates is related to LID [92, 93]. Therefore, there is convincing evidence to further study the role of mGluR5 antagonist in LID treatment. AFQ056 is a novel and effective selective inhibitor of mGluR5 subtype. Two randomized clinical studies involving 59 PD individuals revealed that AFQ056 25-150 mg twice daily for 16 days improved dyskinesia severity scores without exacerbating parkinsonian symptoms [94]. In another dose-finding clinical trial, a total of 197 PD subjects troubled with LID who were not taking Amantadine were randomly divided into 6 groups to receive AFQ056 (20 mg, 50 mg, 100 mg, 150 mg and 200 mg daily) or placebo for 12 weeks. The results found significant improvement on the modified Abnormal Involuntary Movements Scale (mAIMS) in subjects randomized to AFQ056 200 mg per day [95]. A small clinical study including 14 PD individuals showed a slight improvement of mAIMS score in AFQ056 treatment group (200 mg/day) while a slight deterioration of Unified Dyskinesia Rating Scale (UDysRS) parts III and IV score compared to placebo. The results of this study were considered inconclusive due to conflicting results and fewer patients [96]. Two phase II randomized clinical studies of 207 PD patients who received immediate-release AFQ056 200 mg/day or received modified-release AFQ056 300 mg/day or 400 mg/day for 12 weeks both failed to show improvement of dyskinesia [97]. Common adverse events of AFQ056 included hallucinations, fatigue, dizziness and nasopharyngitis. A recent meta-analysis including six randomized clinical studies with a total of 485 PD patients revealed that current evidence did not support the effectiveness of AFQ056 in the management of LID in PD patients and the use of AFQ056 for LID treatment was not recommended [98].

Dipraglurant (ADX48621) is a novel and efficacious negative allosteric modulator of mGluR5. Animal experiments indicated that Dipraglurant mitigated the severity of LID in the macaque PD model in a dose-dependent manner without affecting the efficacy of levodopa [99]. A randomized phase IIa clinical study enrolled 76 patients to explore the effectiveness and tolerability of Dipraglurant [13]. The initial dose of Dipraglurant was 50 mg once a day and increased to 100 mg 3 times a day after 2 weeks. The results showed that Dipraglurant had good safety and tolerability. This study also found that Dipraglurant could mitigate peak dose dyskinesia, and its effect of relieving LID deserves further explorations in larger-scale clinical tests. Unfortunately, the phase IIb study of Dipraglurant did not meet the primary end-point and the program was terminated.

### 5.5 Glutamate Release Inhibitor

Naftazone, a drug widely used to treat varicose veins and venous insufficiency, also presented with some anti-glutamatergic properties. A small study of 7 PD patients showed that Naftazone 120 mg/day for 28 days could improve troublesome dyskinesia compared with placebo [100]. A phase IIa study including 16 PD patients found Naftazone 160 mg/day for 2 weeks was safe and generally well tolerated. Although the levodopa challenge test is well designed and allows low variability in motor symptoms and dyskinesia scores, the test failed to demonstrate the efficacy of Naftazone on LID [101]. Further exploration of Naftazone should be conducted in a larger clinical trial with a longer observation period.

## 6 SEROTONINERGIC AGENTS

5-Hydroxytryptamine (5-HT) receptors regulate a variety of biological and neurological processes, including cognition, learning, memory, emotion, anxiety, appetite, nausea, sleep and thermoregulation [102]. 5-HT neurons in the brain mainly originate from the dorsal raphe nuclei in brainstem with their nerve fibers stimulating various components of the basal ganglion circuit, which acts a pivotal part in regulating the excitability of neurons and modulating behavior and movement of the body (Figs. **1** and **2**) [102]. The regulation of 5-HT system has been widely studied as a potential pathway to alleviate PD psychosis and LID.

### 6.1 5-HT_1A_ Receptor Agonists

Presynaptic 5-HT_1A_ receptor agonists reduce the release of dopamine from serotonergic striatal terminals, potentially reducing dyskinesia [103]. Therefore, some clinically available antidepressants acting on 5-HT_1A_ receptor were evaluated in PD subjects. Buspirone improved dyskinesia without exacerbating PD symptoms in a small study of 10 PD subjects [104]. To date, a phase II clinical trial using Buspirone in PD subjects as adjunctive therapy to Amantadine (https://clinicaltrials.gov. identifier: NCT02589340) was terminated due to slow enrollment. A phase III clinical study on Buspirone in advanced PD individuals has passed its completion date while status was not verified for more than 2 years (https://clinicaltrials.gov. identifier: NCT02617017). A recent clinical study including 21 PD individuals was designed to assess the safety and tolerability of Buspirone for anxiety in PD patients. The study found that Buspirone treatment was intolerable with 7 of 17 patients discontinuing Buspirone treatment before the completion of this 12-week study, and 9 of 17 patients experiencing deterioration in motor function [105]. Sarizotan, a selective 5-HT_1A_ receptor agonist, has been reported to be unable to extend ON time without dyskinesia, and higher dose was related to longer OFF times compared to placebo [106, 107]. Eltoprazine is a selective partial agonist of 5-HT_1A_ and 5-HT_1B_ receptors and has shown anti-dyskinetic effect in animal models of LID [108]. A dose-finding clinical study of 22 PD patients showed that Eltoprazine 5mg/day had a beneficial anti-dyskinetic effect without altering the normal motor response to levodopa [109]. Subsequent phase II clinical study on Eltoprazine has passed its completion date while status was not verified for more than 2 years (https://clinicaltrials.gov. identifier: NCT02439125). JM-010 is a combination of zolmitriptan and buspirone [110]. A 2-way crossover study of 30 PD individuals with moderate to severe LID to assess the efficacy, tolerability, and pharmacokinetics of JM-010 has been completed while the results are still pending (https://clinicaltrials.gov. identifier: NCT02439203). Another placebo-controlled study of JM-010 is still under recruiting (https://clinicaltrials.gov. identifier: NCT03956979). Other two agents based on 5HT_1A_ receptor under preparation for the treatment of LID are NLX-112 [111, 112] and F15599 [113].

### 6.2 5-HT_2A/2C_ Antagonists

Clozapine, a dibenzodiazepine, is weak antagonistic to D2 receptors and has significant activity against dopamine D4 and 5-HT2 receptors, particularly in the treatment of PD at a low dose [114, 115]. Clozapine is the only antipsychotic agent that has been shown in clinical trials to be efficacious for PD patients with mental disorders and without exacerbating motor symptoms in PD patients [116, 117]. The average daily effective dose for PD patients is usually 25-50 mg/day and the therapeutic benefit can be achieved when the daily dose is as low as 6.25 mg [116]. Clinical investigations have also confirmed the efficacy of Clozapine for Parkinson's tremor compared with Pimavanserin [118, 119]. A clinical study involving 38 patients with severe PD found that Clozapine at 25-50 mg/day improved LID in PD patients without worsening PD symptoms [120]. Non-dose-dependent agranulocytosis related to Clozapine may be fatal with an incidence of 0.38% [121] hence frequent blood tests are mandatory to monitor the absolute count of neutrophil, preferably in a specialized center. Other reported adverse effects included seizures [122], postural hypotension and drowsiness [115]. The clinical application of Clozapine is limited considering its side effects.

As a highly selective 5-HT_2A_ inverse agonist with no affinity for 5-HT_2B_ receptors, Pimavanserin is an effective therapy for mental disorders of PD patients [123]. A 6-week, phase III trial containing 199 PD individuals investigated the tolerability and effectiveness of Pimavanserin in PD patients with psychosis [124]. Pimavanserin for 40mg/day reflected a significant improvement of psychosis in subjects with moderate or severe PD, and PD patients in Pimavanserin group also showed improvements in nocturnal sleep and daytime wakefulness compared with placebo [124]. Pimavanserin was also found to be well-tolerated with no deterioration of parkinsonism or other safety concerns. The common adverse reactions of Pimavanserin included nausea, headache, urinary tract infection and peripheral edema [124]. In a randomized clinical study of 383 PD patients, the antipsychotic effect of Pimavanserin was relatively strong in PD patients with cognitive impairment, and its antipsychotic effect could be enhanced by cognitive enhancement drugs [125]. Pimavanserin may benefit patients with PD psychosis, especially for those with few other valuable treatment options [126]. On April 29, 2016, Pimavanserin was approved globally for the first time in the United States for the management of hallucinations and delusions related to PD psychosis [127].

## 7 ACETYLCHOLINE AGENTS

### 7.1 Cholinesterase Inhibitors

Acetylcholine (ACh) produce neurons in the pedunculopontine tegmental nucleus (PPN) and nucleus basalis (Fig. **1**) [128]. PPN is directly involved in the control of gait and posture, while nucleus basalis contributes to the attention process required for these tasks [2, 129, 130]. Therefore, strategies to increase ACh levels in the brain were supposed to alleviate axial motor symptoms of PD. Rivastigmine and donepezil are clinically available cholinesterase inhibitors licensed for the management of cognitive dysfunction or dementia. Clinical studies revealed that Rivastigmine and Donepezil may help prevent falls in PD sufferers.

A phase II clinical study including 114 PD patients without cognitive impairment revealed that Rivastigmine (from 3 mg/day to a target dose of 12 mg/day) for 12 weeks significantly improved step time variability in simple cognitive dual task during normal walking. While for complex cognitive dual tasks, there was no difference in the improvement of step time variability between different groups [131]. A prospective clinical study including 176 PD patients was conducted to evaluate the effects of Rivastigmine (6 mg/day) on cognitive impairment and falls in PD patients. After 12 months, the number of falls per person as well as the incidence of falls in Rivastigmine group was much lower compared to control group. The Montreal Cognitive Assessment (MoCA) score was also significantly higher in the Rivastigmine treatment group than in the control group, indicating that the incidence of falls may be closely associated with the degree of cognitive impairment [132]. A recent study randomized 19 patients with Parkinson's disease dementia (PDD) to transdermal or oral Rivastigmine (9.5 mg/10 cm^2^ daily or 12 mg/day) group. The study found a significant 15.8% reduction in the mean velocity of the center of pressure after 6 months in the transdermal group compared to placebo. However, the difference was not significant in the oral treatment group. This phenomenon may be explained as there were more fallers in the transdermal group than that in the oral group at baseline, potentially meaning a more serious disease. It should also be noted that there was no significant change in total Matisse Dementia Rating Scale scores and the attention subscores [133]. Although Rivastigmine may help improve certain markers of postural instability, the cognitive correlation between gait and posture needs to be further studied. More recently, a multicenter placebo-controlled trial of 168 PD individuals to evaluate whether the treatment of minor visual hallucinations in PD patients with Rivastigmine could delay the progression of psychosis was terminated early due to slow enrollment. Limited data of the study failed to show benefit for cognition and non-motor symptoms in Rivastigmine treatment group [134]. A post hoc analysis of 1047 individuals with PDD showed that Rivastigmine treatment had clinical benefits on cognition function. The study also found that the cognitive benefit of Rivastigmine was greater in PDD patients with OH, which may be mediated by direct antihypertensive effects [135].

Previous clinical studies on Donepezil (5-10 mg) found improvement in cognition, executive function in PDD patients [136]. Recent studies evaluated the efficacy of Donepezil in gait and balance of PD patients. A randomized clinical study of 23 PD individuals with advanced postural instability found that Donepezil 5 mg/day for 6 weeks significantly reduced the incidence of fall compared to placebo [137]. A recent clinical trial including 45 PD patients reported that Donepezil 10 mg/day for 6 weeks failed to improve static or dynamic balance in PD individuals [138]. A phase IV clinical trial exploring the effects of Donepezil on brain networks for gait and postural in PD patients has passed its completion date, while the status has not been verified for more than 3 years (https://clinicaltrials.gov.identifier: NCT03011476). So far, there seem to be conflicting results regarding the role of cholinesterase inhibitors in preventing falls in PD patients and further research is still needed.

Pyridostigmine, a peripheral inhibitor of acetylcholinesterase, was thought to strengthen both limbs of the baroreflex without causing supine hypertension in a study of PD patients with orthostatic hypotension (OH) [139]. A phase II crossover study of 9 participants was conducted to compare the effectiveness of Pyridostigmine and Fludrocortisone on OH control in PD patients. Pyridostigmine was found to have no positive effect on the subjective severity of OH symptoms in PD individuals [140]. There has been no further study on Pyridostigmine for the treatment of OH in PD patients to date.

### 7.2 Acetylcholine Receptor Agents

ACh interacts with two different receptor types: muscarinic and nicotinic. Muscarinic receptors are distributed in the postsynaptic regions of cholinergic interneurons and GABA-containing neurons in striatum, while nicotinic receptors are distributed in presynaptic sites, including nigrostriatal dopaminergic terminals in which the α6β2* subtype appears to modulate dopamine release in the striatum. These receptors are thought to be more selective targets for PD with less possibility of cholinergic side effects.

### 7.3 Nicotinic Receptor Agonists

A growing body of research suggests that nicotine and other agents acting on nicotinic acetylcholine receptors (nAChRs) may be beneficial to motor symptoms of PD patients [104, 141]. A phase II study of 40 PD subjects reported that high dose of nicotine transdermal absorption was tolerable. However, the trial did not find significant improvements in UPDRS motor score among PD subjects who received high doses of nicotine for over 6 months. The improvement of UPDRS-II and UPDRS-IV score, along with a reduction in the dose of levodopa equivalents, showed possible benefits in patients receiving nicotine therapy, while it should be confirmed in larger clinical trials [142]. Common adverse reactions were nausea and dizziness. A post-hoc analysis of phase I/II study involving 65 PD subjects treated with nicotine tablets (NC001or NP002) for LID found that nicotine tablets administered for 10 weeks significantly improved FOG and falls in PD individuals compared to placebo [143]. A pilot clinical trial of 6 patients to explore the pulsatile delivery of trans-nasal nicotine was already completed, while the result was still pending (https://clinicaltrials.gov. identifier: NCT03865121).

As a novel selective α7 nAChR partial agonist, AQW051 has been proved to effectively reduce the expression of LID in PD animal models [144]. A phase II clinical study involving 67 patients reported that AQW051 administered 10 or 50 mg once daily for 28 days failed to reduce LID and parkinsonian severity measured by mAIMS and UPDRS part III scores, while post hoc exploratory analysis found a possible cognitive benefit from AQW051. AQW051 at 10 or 50 mg once daily was safe and well tolerated, with common adverse effects including fatigue, nausea, dyskinesia, and falls [145].

### 7.4 Muscarinic Receptor Antagonists

Glycopyrrolate is a competitive muscarinic receptor antagonist and is currently available for the treatment of sialorrhea. A randomized clinical study of 23 PD individuals found that Glycopyrrolate 1 mg three times a day for 4 weeks significantly reduced the mean sialorrhea score compared to placebo [146]. A recent 12-week clinical trial of 28 PD subjects showed that Glycopyrrolate was effective in controlling sialorrhea-related disability in PD patients, with the most common adverse effect being dry mouth [147].

Tropicamide is a short acting muscarinic receptor antagonist with a similar pharmacodynamic characteristic to Atropine but a better tolerance. A pilot clinical trial of 19 PD subjects reported that intra-oral slow dissolving thin films containing tropicamide (NH004) were safe and efficient for the treatment of sialorrhea [148]. However, there was no other further exploration on the anti-sialorrhea effects of NH004.

### 7.5 Botulinum Toxin

Botulinum toxin (BoNT) reduces cholinergic effects by decreasing the release of acetylcholine in the muscle endplate. Focal injection of BoNT is the most effective therapy for focal dystonia, blepharospasm as well as eyelid apraxia in PD patients (level A recommendation) [149].

Several studies have explored the efficacy of BoNT injection in controlling PD tremor. A 38-week clinical study evaluated 28 PD individuals who received upper limbs injection of BoNT-A at weeks 0, 16 and 32. The study found a significant reduction in the severity of rest tremor (UPDRS item 20) in treated arms from 2.7 ± 0.6 at week 0 to 2.0 ± 0.8 at week 16 and to 2.1 ± 0.7 at week 32. Action tremor (UPDRS item 21) also improved while the difference was not statistically significant. Reported adverse effects included mild muscle weakness without interfering with daily life [150]. A phase II clinical study of 30 PD patients was conducted to explore the tolerability and effectiveness of BoNT-A injection for PD tremor. The results showed that clinical rating scores of rest tremor and tremor severity were significantly ameliorated at 4 and 8 weeks after injection of BoNT-A, action tremor was also significantly improved at 8 weeks, the incidence of significant hand weakness was low [151].

A randomized clinical study of 12 advanced PD patients to assess the efficacy of BoNT-A injection for limb pain in advanced PD has been completed. The results found that BoNT-A injection significantly reduced the numeric rating scale (NRS) score in PD patients at week 4 while the difference was not statistically significant compared with placebo. PD patients with dystonia pain showed a greater decrease in NRS score after 4 weeks of treatment of BoNT-A injection. Further studies may focus on evaluating the efficacy of BoNT-A, especially for dystonia pain [152].

BoNT injection was thought to be an effective therapy for overactive bladder in PD patients [149]. A phase IV clinical study of 10 PD patients with neurogenic lower urinary tract dysfunction found that 200 IU BoNT-A injection was a safe and efficient therapy for PD patients with neurogenic lower urinary tract dysfunction. Urodynamic, bladder diary parameters and international consultation on incontinence questionnaire in PD subjects were improved after 200 IU BoNT-A injections [153]. A recent clinical study of 24 PD individuals reported that intradetrusor injection of BoNT-A 100 U is an efficient and safe therapy for PD patients with overactive bladder symptoms [154]. Further research was still needed to confirm the importance of these findings.

Recently, BoNT injection has been shown to be an effective and safe therapy for sialorrhea in PD patients [149, 155, 156]. An 8-year follow-up study of 65 patients (33 PD patients and 32 amyotrophic lateral scleroses) with severe sialorrhea and received at least two ultrasound-guided intrasalivary glands abobotulinumtoxinA 250 U or rimabotulinumtoxinB 2500 U injections found that both serotypes produced a significant beneficial effect in 89% of treatments compare to baseline, with a mean benefit period of 87 days and PD subjects showed a longer duration of benefit [157]. A phase III study of 184 patients (130 PD patients) found a significant reduction in mean unstimulated salivary flow rate at week 4 after incobotulinumtoxin A 100 U injection compared to placebo. Incobotulinumtoxin A 100 U is efficacious and safe therapy for adult chronic sialorrhea [158]. Data from a clinical study of 173 patients (123 PD patients) supported the long-term efficacy and safety of repeated incobotulinumtoxinA treatment for sialorrhea over 64 weeks, with common adverse events being dysphagia and dry mouth [159]. Recently, another clinical study containing 122 PD patients found that injection of rimabotulinumtoxinB (2500 U and 3500 U) in adults was well tolerated and significantly improved unstimulated salivary flow rate at week 4 compared to placebo. The therapeutic effect appeared as early as 1 week after injection and remained for about 13 weeks over the treatment cycle [160].

## 8 ADRENERGIC RECEPTOR AGENTS

Noradrenergic neurotransmission in the locus coeruleus plays a part in the pathophysiology of FOG and akinetic-rigid phenotype in PD by influencing the release of dopamine and glutamate (Figs. **1** and **2**) [161]. Supplementation of norepinephrine with selective adrenergic receptor agonists, presynaptic α2 adrenergic receptor antagonists or with noradrenergic reuptake inhibitors such as methylphenidate may improve FOG and motor symptoms in PD patients [162].

### 8.1 Adrenergic Receptor Agonists

Droxidopa is a precursor of noradrenaline that has been used for the treatment of neurogenic OH. A small exploratory study of 51 PD subjects designed to investigate the efficacy of Droxidopa for controlling neurogenic OH in PD patients found that the orthostatic hypotension questionnaire composite score showed no beneficial change compared to placebo at week 8. However, the study found improvement of dizziness, reduction in falls and lightheadedness, deserving further study in larger clinical trials [163]. Another study of 171 PD individuals found an improvement in the primary neurogenic OH symptoms of dizziness/lightheadedness from baseline to week 1 as measured by Orthostatic Hypotension Symptom Assessment (OHSA) item 1 [164]. Therefore, the study provided evidence for the short-term efficacy of Droxidopa for neurogenic OH in PD patients. Studies also reported that droxidopa reduced falls in PD patients with neurogenic OH, and authors attributed the reduction in falls in Droxidopa treatment group to the improvement of OH, which should be confirmed in larger clinical trials [165, 166]. Droxidopa was well-tolerated without significant adverse reactions. A randomized clinical study of 219 PD individuals reported improvements in motor function and activities of daily living in patients with moderate to severe PD who received 600 mg/day of Droxidopa as an adjunct to levodopa and/or DA for 8 weeks [167]. A double-blind clinical study involving 21 subjects was designed to evaluate the effectiveness of Droxidopa on gait speed, kyphosis, and functional reach of PD patients. Medication was titrated from 300 mg/day to 1800 mg/day until it was intolerable. The preliminary results showed that there was no significant improvement in gait and posture.

### 8.2 α2-adrenergic Receptor Antagonist

α2-noradrenergic receptors are strongly expressed in GABA neurons of striatum and promote involuntary movement. Several compounds targeting α2-noradrenergic receptors were studied for the suppression of LID [168]. Fipamazole is a novel non- selective α2-adrenergic receptor antagonist. An investigation of 10 subjects who took Fipamazole at doses up to 90 mg showed good tolerance and inhibition of LID without aggravating parkinsonism. A 28-day multicenter trial involving 179 subjects with advanced PD was designed to assess the efficacy of Fipamazole against LID [11]. The initial doses of Fipamazole were increased from 30 mg/day to 90 mg/day, 180 mg/day, and 270 mg/day. The results found that 90 mg/day group could significantly reduce LID. The adverse reaction was a slight increase in blood pressure, while the patients showed good tolerance [166].

### 8.3 Noradrenergic Reuptake Inhibitor

Methylphenidate blocks the reuptake of dopamine and norepinephrine by inhibiting presynaptic dopamine and norepinephrine transporters and has been investigated for gait therapy in PD with inconsistent results. A randomized clinical trial involving 17 PD patients reported that Methylphenidate at 80 mg/day for 12 weeks failed to improve gait while tended to exacerbate motor function, sleepiness, and quality of life [169]. A multicenter clinical trial of 65 PD patients with severe gait disorders or FOG found that Methylphenidate improved FOG and gaits in advanced PD patients who received STN-DBS [170]. Another clinical study investigated 24 PD patients who were randomly assigned to Methylphenidate (1 mg/kg/D) or placebo in a 1:1 ratio for 3 months. The primary outcome was that Methyl-phenidate failed to improve gait hypokinesia in patients who received dopaminergic treatment, and FOG severity was not improved in Methylphenidate treatment group compared to placebo [171].

## 9 GABA RECEPTOR AGONISTS

GABA is the main inhibitory neurotransmitter in the central nervous system and is assumed to be the pathophysiological basis of PD (Figs. **1** and **2**) [63]. Owing to the extensive distribution of the GABA receptors in the non-basal ganglia areas, targeting GABA receptors is still not an effective method to alleviate PD symptoms. However, recent studies suggest that selective regulation of GABA receptors may improve motor symptoms in PD patients [172].

Zolpidem is an imidazopyridine agent which acts on GABA receptor and is commonly used for the treatment of insomnia [173]. Preclinical study showed that Zolpidem ameliorates motor impairments in PD animal models, and Zolpidem sensitive GABA_A_ receptor may be a new target for the treatment of motor symptoms of PD. Zolpidem has been reported to have unexpected effects on motor dysfunction and neuropsychiatric symptoms in PD patients [174-176]. A phase II study aiming to investigate the effectiveness of Zolpidem on motor function of PD has been completed while the results are still pending. (https://clinicaltrials.gov. identifier: NCT03621046).

Zulanolone is an oral neuroactive steroid GABA_A_ receptor positive allosteric modulator and has been studied for its tolerability and effectiveness in PD patients. An open label exploratory study involving 14 subjects showed that Zulanolone as an adjuvant to dopaminergic therapy for 7 days could improve tremor in PD patients compared to baseline values measured by the UPDRS Part II/III total tremor score. In addition, Zulanolone treatment improved overall motor symptoms of PD patients and motor and non-motor experiences in daily life. Common adverse reactions were sedation, drowsiness as well as dizziness [177]. Due to the limited number of PD patients and short duration, Zulanolone for the treatment of tremor in PD patients deserves further study. A phase II study evaluating the efficacy and tolerability of Zulanolone on motor symptoms of PD has been completed while the results are still pending (https://clinicaltrials.gov. identifier: NCT03000569).

## 10 CANNABINOID RECEPTOR AGONISTS

Cannabinoid acts primarily through cannabinoid type 1 (CB1) receptors in the central nervous system. CB1 receptors are widely distributed in the basal ganglia area in which endogenous cannabinoids exert complex effects on motor activity through interactions with dopamine, glutamate, GABA and other neurotransmitters (Fig. **1**) [178]. Several studies have assessed the therapeutic potential of Cannabinoid for PD patients.

So far, two old studies on Cannabinoid therapy for dyskinesia have shown conflicting results [179, 180]. Oral Cannabinoid was not considered to be helpful in LID treatment due to the inconsistent results of the two studies [181]. An ongoing phase II randomized clinical trial of 60 PD patients will evaluate tolerance and efficacy of Cannabinoid on motor symptoms in PD patients, with efficacy of Cannabinoid on dyskinesia to be a possible secondary outcome indicator (https://clinicaltrials.gov.identifier: NCT03582137). A recent randomized clinical study involving 24 PD individuals found that acute administration of Cannabidiol 300 mg reduced anxiety in PD patients induced by a simulated public speaking test and reduced the amplitude of tremor in anxiogenic situations [182]. The results should be confirmed in a larger clinical study. A phase II clinical study of 13 PD patients received a purified form of Cannabinoid, GWP42003-P oral solution for 20 mg/kg/day. The preliminary results showed that a total of 10 patients completed the clinical study. After 5 weeks of medication, the total UPDRS score reduced by an average of 7.7 points and the UPDRS III tremor score decreased by 0.4 points on average. Common adverse reactions include fatigue, weight gain, diarrhea, abdominal pain, drowsiness, and dizziness (https://clinicaltrials.gov. identifier: NCT02818777). In summary, the application of Cannabinoid in PD still needs further exploration.

## 11 HISTAMINE RECEPTOR ANTAGONISTS

Histaminergic innervation of basal ganglia plays an important part in regulating the release of glutamate, GABA, dopamine, and acetylcholine through abundant inhibitory presynaptic H3 receptors and excitatory postsynaptic H2 receptors in the striatum (Fig. **1**). Animal studies suggested that histamine H2 and H3 receptors may be potential targets for LID treatment [183, 184].

Famotidine is a selective histamine H_2_ receptor antagonist, which is applicable to various gastrointestinal conditions. Preclinical study also found that famotidine enhanced the anti-parkinsonian effects of levodopa and reduced peak-dose LID without producing disabling dyskinesia. However, a proof-of-concept study including 7 PD individuals failed to find that Famotidine reduced LID. There was no significant improvement in UDysRS part III, UDysRS part I and UDysRS part II scores compared with placebo. Famotidine was well tolerated without significant adverse effects [185]. To date, there has been no further study on famotidine in the treatment of LID.

## 12 OPIOIDS

Opioids are well known to play an indispensable part in the pathophysiology of pain. Recent studies have assessed the effectiveness of opioid preparations on PD-related pain. A phase II clinical study of 202 PD patients with chronic or severe pain aimed to access the analgesic effects of oxycodone–naloxone prolonged-release (OXN PR), a combination of oxycodone (opioid analgesic) and naloxone (opioid receptor antagonist). The results showed that OXN PR failed to improve mean 24-h pain scores at 16 weeks. However, the per-protocol analysis found that appropriate adherence led to significant improvement of mean 24-h pain scores at 16 weeks in OXN PR treatment group compared to placebo [186]. Common adverse events of OXN PR are constipation and nausea. A small clinical study of 16 PD subjects showed significant pain relief at a low dose of OXN-PR, as measured by numerical rating scale and brief pain inventory (BPI) score [187]. The efficacy of OXN PR on PD related pain needs to be further investigated in large clinical trials.

Opioid-binding agents may also be beneficial to the management of impulse control disorders (ICDs). A recent study of 50 PD patients with ICDs aimed to investigate the clinical efficacy of Naltrexone, a nonselective, competitive opioid receptor antagonist in the treatment of ICDs. The study revealed that naltrexone for 50-100 mg/day failed to alleviate the severity of ICDs in PD patients using a clinical global impression scale. However, results from a PD-specific ICD rating scale supported further research on naltrexone for ICDs treatment in PD patients [188].

## CONCLUSION

A series of nondopaminergic agents and candidate compounds have been developed or under development mainly targets motor fluctuations and dyskinesia in PD individuals. Adenosine A2A receptor antagonist Istradefylline, mixed MAO-B inhibitor and glutamate release inhibitors, Zonisamide and Safinamide were licensed for the management of motor fluctuations in PD patients. Long-acting glutamate receptor antagonist ADS-5102 improved LID in PD patients while adverse effects should be monitored. Although meta-botropic glutamate receptor targets seem to have strong scientific validity for PD, different trials showed that both mGluR5 antagonists and mGluR4 positive allosteric modulators have minimal or no effect on LID. The clinical study of other glutamatergic agents, such as Topiramate, Dextromethorphan, Memantine and Naloxone have not shown significant clinical efficacy, and there was no further development or research planned. Preliminary studies found that 5HT_1A_ agonists Buspirone and Eltoprazine showed clinical benefits to dyskinesia and further studies are underway. Studies have consistently linked smoking and coffee drinking to a lower risk of PD, while recent studies on Caffeine and Nicotine failed to show overall benefits for motor symptoms of PD patients. GABA_A_ receptor positive allosteric modulator Zulanolone showed clinical efficacy to tremor and overall motor symptoms of PD, meriting further study. Off-label use of Rivastigmine revealed clinical efficacy to gait and balance in PD patients. However, the effects of Donepezil and Methylphenidate on gait and balance are inconsistent.

Concomitantly, studies also evaluated nondopaminergic agents as treatment options for non-motor symptoms in PD patients. Istradefylline as an adenosine A2A receptor antagonist improved daytime sleepiness, apathy, depression, and lower urinary tract symptoms in PD patients. Pimavanserin acting on 5-HT_2A_ receptor was approved globally for the management of hallucinations and delusions related to PD psychosis. According to recent studies, Droxidopa mainly targets adrenergic receptor should be a potential option for the management of OH in PD patients, although the benefits were short-term. Highly selective MAO-B inhibitor Safinamide showed prominent clinical beneficial effects on chronic pain, executive function, depression, sleep, and mood disorders of PD individuals. As a clinically available MAO-B inhibitor, Zonisamide also showed clinical efficacy for depressive symptoms and sleep disorders in PD patients with tremor. Clinical use of muscarinic receptor antagonists, such as Glycopyrrolate and Tropicamide improved sialorrhea in PD patients without causing central adverse effects. Focally injection of BoNT remains an effective therapeutic option for focal dystonia, blepharospasm, bladder overactivity, sialorrhea and eyelid apraxia in PD patients. Opioid-binding agents may be beneficial to the pain control and impulse control disorders in PD patients while requiring further exploration.

In conclusion, nondopaminergic preparations continue to be extensively researched and remain a useful treatment option for motor and nonmotor symptoms of PD patients. To date, no single nondopaminergic drug can replace levodopa, considering efficacy and tolerance. The best use of nondopaminergic medication appears to be a promising strategy to reduce the required levodopa doses thus motor complications of dopamine replacement therapy, as well as symptoms that nondopaminergic systems may mediate.

## LIST OF ABBREVIATIONS

5-HT: 5-Hydroxytryptamine
ACh: Acetylcholine
AD: Alzheimer Disease
AMPA: α-amino-3-hydroxy-5-methyl-4-isoxazole propionic
BoNT: Botulinum Toxin
BPI: Brief Pain Inventory
COMT: Catechol-O-methyltransferase
DA: Dopamine Receptor Agonists
DLB: Dementia with Lewy Bodies
FOG: Freezing of Gait
GABA: γ-aminobutyric Acid
ICDs: Impulse Control Disorders
iGluRs: Inotropic Glutamate Receptors
IR: Immediate Release
LID: Levodopa Induced Dyskinesia
mAIMS: Modified Abnormal Involuntary Movements Scale
MAO-B: Monoamine Oxidase-B
mGluRs: Metabotropic Glutamate Receptors
MoCA: Montreal Cognitive Assessment
nAChRs: Nicotinic Acetylcholine Receptors
NMDA: N-methyl-D-Aspartate
NRS: Numeric Rating Scale
OH: Orthostatic Hypotension
PD: Parkinson's Disease
PDD: Parkinson's Disease Dementia
PPN: Pedunculopontine Tegmental Nucleus
STN-DBS: Subthalamic Nucleus Deep Brain Stimulation
UDysRS: Unified Dyskinesia Rating Scale
UPDRS: Unified Parkinson’s Disease Rating Scale

## References

[1] Armstrong M.J., Okun M.S. (2020). Diagnosis and treatment of parkinson disease: A review.. JAMA.

[2] Kalia L.V., Brotchie J.M., Fox S.H. (2013). Novel nondopaminergic targets for motor features of Parkinson’s disease: Review of recent trials.. Mov. Disord..

[3] Müller T. (2020). Pharmacokinetics and pharmacodynamics of levodopa/carbidopa cotherapies for Parkinson’s disease.. Expert Opin. Drug Metab. Toxicol..

[4] Müller T. (2013). Detoxification and antioxidative therapy for levodopa-induced neurodegeneration in Parkinson’s disease.. Expert Rev. Neurother..

[5] Chaudhuri K.R., Todorova A., Nirenberg M.J., Parry M., Martin A., Martinez-Martin P., Rizos A., Henriksen T., Jost W., Storch A., Ebersbach G., Reichmann H., Odin P., Antonini A. (2015). A pilot prospective, multicenter observational study of dopamine agonist withdrawal syndrome in Parkinson’s disease.. Mov. Disord. Clin. Pract. (Hoboken).

[6] Baig F., Kelly M.J., Lawton M.A., Ruffmann C., Rolinski M., Klein J.C., Barber T., Lo C., Ben-Shlomo Y., Okai D., Hu M.T. (2019). Impulse control disorders in Parkinson disease and RBD: A longitudinal study of severity.. Neurology.

[7] Solla P., Fasano A., Cannas A., Marrosu F. (2015). Suicide and dopamine agonist withdrawal syndrome in Parkinson’s disease.. Mov. Disord..

[8] Pinna A. (2014). Adenosine A2A receptor antagonists in Parkinson’s disease: progress in clinical trials from the newly approved istradefylline to drugs in early development and those already discontinued.. CNS Drugs.

[9] Elkouzi A., Vedam-Mai V., Eisinger R.S., Okun M.S. (2019). Emerging therapies in Parkinson disease - repurposed drugs and new approaches.. Nat. Rev. Neurol..

[10] Charvin D., Medori R., Hauser R.A., Rascol O. (2018). Therapeutic strategies for Parkinson disease: Beyond dopaminergic drugs.. Nat. Rev. Drug Discov..

[11] Lewitt P.A., Hauser R.A., Lu M., Nicholas A.P., Weiner W., Coppard N., Leinonen M., Savola J.M. (2012). Randomized clinical trial of fipamezole for dyskinesia in Parkinson disease (FJORD study).. Neurology.

[12] Vijverman A-C., Fox S.H. (2014). New treatments for the motor symptoms of Parkinson’s disease.. Expert Rev. Clin. Pharmacol..

[13] Tison F., Keywood C., Wakefield M., Durif F., Corvol J.C., Eggert K., Lew M., Isaacson S., Bezard E., Poli S.M., Goetz C.G., Trenkwalder C., Rascol O. (2016). A phase 2A trial of the novel mGluR5-negative allosteric modulator dipraglurant for levodopa-induced dyskinesia in Parkinson’s disease.. Mov. Disord..

[14] Hauser R.A., Pahwa R., Tanner C.M., Oertel W., Isaacson S.H., Johnson R., Felt L., Stempien M.J. (2017). ADS-5102 (Amantadine) extended-release capsules for levodopa-induced dyskinesia in Parkinson’s disease (EASE LID 2 study): Interim results of an open-label safety study.. J. Parkinsons Dis..

[15] Müller T. (2020). Safinamide in the treatment of Parkinson’s disease.. Neurodegener. Dis. Manag..

[16] Morelli M., Wardas J. (2001). Adenosine A(2a) receptor antagonists: Potential therapeutic and neuroprotective effects in Parkinson’s disease.. Neurotox. Res..

[17] Gołembiowska K., Dziubina A. (2012). The effect of adenosine A(2A) receptor antagonists on hydroxyl radical, dopamine, and glutamate in the striatum of rats with altered function of VMAT2.. Neurotox. Res..

[18] Hazama T., Fukada K., Mitani Y., Kinoshita M., Takata K., Kokunai Y., Sawada J-i. (2016). Clinical characteristics of Parkinson’s disease patients responsive to Istradefylline treatment.. Parkinsonism Relat. Disord..

[19] Mizuno Y., Kondo T. (2013). Adenosine A2A receptor antagonist istradefylline reduces daily OFF time in Parkinson’s disease.. Mov. Disord..

[20] Kondo T., Mizuno Y. (2015). A long-term study of istradefylline safety and efficacy in patients with Parkinson disease.. Clin. Neuropharmacol..

[21] Iijima M., Orimo S., Terashi H., Suzuki M., Hayashi A., Shimura H., Mitoma H., Kitagawa K., Okuma Y. (2019). Efficacy of istradefylline for gait disorders with freezing of gait in Parkinson’s disease: A single-arm, open-label, prospective, multicenter study.. Expert Opin. Pharmacother..

[22] Hattori N., Kitabayashi H., Kanda T., Nomura T., Toyama K., Mori A. (2020). A pooled analysis from phase 2b and 3 studies in Japan of Istradefylline in Parkinson’s disease.. Mov. Disord..

[23] Kitta T., Yabe I., Takahashi I., Matsushima M., Sasaki H., Shinohara N. (2016). Clinical efficacy of istradefylline on lower urinary tract symptoms in Parkinson’s disease.. Int. J. Urol..

[24] Suzuki K., Miyamoto M., Miyamoto T., Uchiyama T., Watanabe Y., Suzuki S., Kadowaki T., Fujita H., Matsubara T., Sakuramoto H., Hirata K. (2017). Istradefylline improves daytime sleepiness in patients with Parkinson’s disease: An open-label, 3-month study.. J. Neurol. Sci..

[25] Nagayama H., Kano O., Murakami H., Ono K., Hamada M., Toda T., Sengoku R., Shimo Y., Hattori N. (2019). Effect of istradefylline on mood disorders in Parkinson’s disease.. J. Neurol. Sci..

[26] Shimo Y., Maeda T., Chiu S.W., Yamaguchi T., Kashihara K., Tsuboi Y., Nomoto M., Hattori N., Watanabe H., Saiki H. (2021). Influence of istradefylline on non-motor symptoms of Parkinson’s disease: A subanalysis of a 1-year observational study in Japan (J-FIRST).. Parkinsonism Relat. Disord..

[27] Müller T., Öhm G., Eilert K., Möhr K., Rotter S., Haas T., Küchler M., Lütge S., Marg M., Rothe H. (2017). Benefit on motor and non-motor behavior in a specialized unit for Parkinson’s disease.. J. Neural Transm. (Vienna).

[28] Salamone J.D. (2010). Preladenant, a novel adenosine A(2A) receptor antagonist for the potential treatment of parkinsonism and other disorders.. IDrugs.

[29] Hodgson R.A., Bedard P.J., Varty G.B., Kazdoba T.M., Di Paolo T., Grzelak M.E., Pond A.J., Hadjtahar A., Belanger N., Gregoire L., Dare A., Neustadt B.R., Stamford A.W., Hunter J.C. (2010). Preladenant, a selective A(2A) receptor antagonist, is active in primate models of movement disorders.. Exp. Neurol..

[30] Hauser R.A., Cantillon M., Pourcher E., Micheli F., Mok V., Onofrj M., Huyck S., Wolski K. (2011). Preladenant in patients with Parkinson’s disease and motor fluctuations: A phase 2, double-blind, randomised trial.. Lancet Neurol..

[31] Hattori N., Kikuchi M., Adachi N., Hewitt D., Huyck S., Saito T. (2016). Adjunctive preladenant: A placebo-controlled, dose-finding study in Japanese patients with Parkinson’s disease.. Parkinsonism Relat. Disord..

[32] Hauser R.A., Stocchi F., Rascol O., Huyck S.B., Capece R., Ho T.W., Sklar P., Lines C., Michelson D., Hewitt D. (2015). Preladenant as an adjunctive therapy with levodopa in Parkinson disease: Two randomized clinical trials and lessons learned.. JAMA Neurol..

[33] Stocchi F., Rascol O., Hauser R.A., Huyck S., Tzontcheva A., Capece R., Ho T.W., Sklar P., Lines C., Michelson D., Hewitt D.J. (2017). Randomized trial of preladenant, given as monotherapy, in patients with early Parkinson disease.. Neurology.

[34] Hauser R.A., Olanow C.W., Kieburtz K.D., Pourcher E., Docu-Axelerad A., Lew M., Kozyolkin O., Neale A., Resburg C., Meya U., Kenney C., Bandak S. (2014). Tozadenant (SYN115) in patients with Parkinson’s disease who have motor fluctuations on levodopa: A phase 2b, double-blind, randomised trial.. Lancet Neurol..

[35] Noyce A.J., Bestwick J.P., Silveira-Moriyama L., Hawkes C.H., Giovannoni G., Lees A.J., Schrag A. (2012). Meta-analysis of early nonmotor features and risk factors for Parkinson disease.. Ann. Neurol..

[36] Altman R.D., Lang A.E., Postuma R.B. (2011). Caffeine in Parkinson’s disease: A pilot open-label, dose-escalation study.. Mov. Disord..

[37] Postuma R.B., Lang A.E., Munhoz R.P., Charland K., Pelletier A., Moscovich M., Filla L., Zanatta D., Rios Romenets S., Altman R., Chuang R., Shah B. (2012). Caffeine for treatment of Parkinson disease: A randomized controlled trial.. Neurology.

[38] Postuma R.B., Anang J., Pelletier A., Joseph L., Moscovich M., Grimes D., Furtado S., Munhoz R.P., Appel-Cresswell S., Moro A., Borys A., Hobson D., Lang A.E. (2017). Caffeine as symptomatic treatment for Parkinson disease (Café-PD): A randomized trial.. Neurology.

[39] Müller T., Möhr J.D. (2019). Pharmacokinetics of monoamine oxidase B inhibitors in Parkinson’s disease: Current status.. Expert Opin. Drug Metab. Toxicol..

[40] Mizuno Y., Kondo T., Kuno S., Nomoto M., Yanagisawa N. (2010). Early addition of selegiline to L-Dopa treatment is beneficial for patients with Parkinson disease.. Clin. Neuropharmacol..

[41] Teo K.C., Ho S.L. (2013). Monoamine oxidase-B (MAO-B) inhibitors: Implications for disease-modification in Parkinson’s disease.. Transl. Neurodegener..

[42] Blair H.A., Dhillon S. (2017). Safinamide: A Review in Parkinson’s Disease.. CNS Drugs.

[43] Stocchi F., Borgohain R., Onofrj M., Schapira A.H., Bhatt M., Lucini V., Giuliani R., Anand R. (2012). A randomized, double-blind, placebo-controlled trial of safinamide as add-on therapy in early Parkinson’s disease patients.. Mov. Disord..

[44] Borgohain R., Szasz J., Stanzione P., Meshram C., Bhatt M., Chirilineau D., Stocchi F., Lucini V., Giuliani R., Forrest E., Rice P., Anand R. (2014). Randomized trial of safinamide add-on to levodopa in Parkinson’s disease with motor fluctuations.. Mov. Disord..

[45] Borgohain R., Szasz J., Stanzione P., Meshram C., Bhatt M.H., Chirilineau D., Stocchi F., Lucini V., Giuliani R., Forrest E., Rice P., Anand R. (2014). Two-year, randomized, controlled study of safinamide as add-on to levodopa in mid to late Parkinson’s disease.. Mov. Disord..

[46] Cattaneo C., Müller T., Bonizzoni E., Lazzeri G., Kottakis I., Keywood C. (2017). Long-term effects of safinamide on mood fluctuations in Parkinson’s disease.. J. Parkinsons Dis..

[47] Cattaneo C., Jost W.H., Bonizzoni E. (2020). Long-term efficacy of safinamide on symptoms severity and quality of life in fluctuating Parkinson’s disease patients.. J. Parkinsons Dis..

[48] Cattaneo C., Kulisevsky J., Tubazio V., Castellani P. (2018). Long-term efficacy of safinamide on Parkinson’s disease chronic pain.. Adv. Ther..

[49] Santos García D., Labandeira Guerra C., Yáñez Baña R., Cimas Hernando M.I., Cabo López I., Paz Gonález J.M., Alonso Losada M.G., González Palmás M.J., Martínez Miró C. (2021). Safinamide improves non-motor symptoms burden in Parkinson’s disease: An open-label prospective study.. Brain Sci..

[50] Santos García D., Yáñez Baña R., Labandeira Guerra C., Cimas Hernando M.I., Cabo López I., Paz González J.M., Alonso Losada M.G., Gonzalez Palmás M.J., Cores Bartolomé C., Martínez Miró C. (2021). Pain improvement in Parkinson’s disease patients treated with safinamide: Results from the SAFINONMOTOR study.. J. Pers. Med..

[51] Rinaldi D., Sforza M., Assogna F., Savini C., Salvetti M., Caltagirone C., Spalletta G., Pontieri F.E. (2021). Safinamide improves executive functions in fluctuating Parkinson’s disease patients: an exploratory study.. J. Neural Transm. (Vienna).

[52] Peña E., Borrué C., Mata M., Martínez-Castrillo J.C., Alonso-Canovas A., Chico J.L., López-Manzanares L., Llanero M., Herreros-Rodríguez J., Esquivel A., Maycas-Cepeda T., Ruíz-Huete C. (2021). Impact of safinamide on depressive symptoms in Parkinson’s disease patients (SADness-PD study): A multicenter retrospective study.. Brain Sci..

[53] Labandeira C.M., Alonso Losada M.G., Yáñez Baña R., Cimas Hernando M.I., Cabo López I., Paz González J.M., Gonzalez Palmás M.J., Martínez Miró C., Santos García D. (2021). Effectiveness of safinamide over mood in Parkinson’s disease patients: Secondary analysis of the open-label study SAFINONMOTOR.. Adv. Ther..

[54] Deeks E.D. (2015). Safinamide: First global approval.. Drugs.

[55] Murata M., Hasegawa K., Kanazawa I. (2007). Zonisamide improves motor function in Parkinson disease: A randomized, double-blind study.. Neurology.

[56] Maeda T., Takano D., Yamazaki T., Satoh Y., Nagata K. (2015). Zonisamide in the early stage of Parkinson’s disease.. Neurol. Clin. Neurosci..

[57] Murata M., Hasegawa K., Kanazawa I., Shirakura K., Kochi K., Shimazu R., Kimura T., Yoshida K., Abe T., Kurita K., Yoshizawa K., Tamaoka A., Nakano I., Shimizu T., Hattori N., Mizusawa H., Kuno S., Yokochi F., Hirabayashi K., Horiuchi E., Kawashima N., Koike R., Ishikawa A., Kuriyama M., Mizoguchi K., Mitake S., Washimi Y., Tatsuoka Y., Fujimura H., Toda K., Kondo T., Nakashima K., Nomoto M., Uozumi T., Sato A., Matsuo H., Tsuruta K. (2015). Randomized placebo‐controlled trial of zonisamide in patients with Parkinson’s disease.. Neurol. Clin. Neurosci..

[58] Murata M., Hasegawa K., Kanazawa I., Fukasaka J., Kochi K., Shimazu R. (2015). Zonisamide improves wearing-off in Parkinson’s disease: A randomized, double-blind study.. Mov. Disord..

[59] Murata M., Odawara T., Hasegawa K., Iiyama S., Nakamura M., Tagawa M., Kosaka K. (2018). Adjunct zonisamide to levodopa for DLB parkinsonism: A randomized double-blind phase 2 study.. Neurology.

[60] Murata M., Odawara T., Hasegawa K., Kajiwara R., Takeuchi H., Tagawa M., Kosaka K. (2020). Effect of zonisamide on parkinsonism in patients with dementia with Lewy bodies: A phase 3 randomized clinical trial.. Parkinsonism Relat. Disord..

[61] Hasegawa K., Kochi K., Maruyama H., Konishi O., Toya S., Odawara T. (2021). Efficacy and safety of zonisamide in dementia with lewy bodies patients with parkinsonism: A post hoc analysis of two randomized, double-blind, placebo-controlled trials.. J. Alzheimers Dis..

[62] Suzuki K., Fujita H., Matsubara T., Haruyama Y., Kadowaki T., Funakoshi K., Watanabe Y., Hirata K. (2021). Zonisamide effects on sleep problems and depressive symptoms in Parkinson’s disease.. Brain Behav..

[63] Jamwal S., Kumar P. (2019). Insight into the emerging role of striatal neurotransmitters in the pathophysiology of Parkinson’s disease and Huntington’s disease: A review.. Curr. Neuropharmacol..

[64] Sebastianutto I., Cenci M.A. (2018). mGlu receptors in the treatment of Parkinson’s disease and L-DOPA-induced dyskinesia.. Curr. Opin. Pharmacol..

[65] Zhang Z., Zhang S., Fu P., Zhang Z., Lin K., Ko J.K., Yung K.K. (2019). Roles of glutamate receptors in Parkinson’s disease.. Int. J. Mol. Sci..

[66] Reiner A., Levitz J. (2018). Glutamatergic signaling in the central nervous system: Ionotropic and metabotropic receptors in concert.. Neuron.

[67] Sawada H., Oeda T., Kuno S., Nomoto M., Yamamoto K., Yamamoto M., Hisanaga K., Kawamura T. (2010). Amantadine for dyskinesias in Parkinson’s disease: A randomized controlled trial.. PLoS One.

[68] Goetz C.G., Stebbins G.T., Chung K.A., Hauser R.A., Miyasaki J.M., Nicholas A.P., Poewe W., Seppi K., Rascol O., Stacy M.A., Nutt J.G., Tanner C.M., Urkowitz A., Jaglin J.A., Ge S. (2013). Which dyskinesia scale best detects treatment response?. Mov. Disord..

[69] Chan H.F., Kukkle P.L., Merello M., Lim S.Y., Poon Y.Y., Moro E. (2013). Amantadine improves gait in PD patients with STN stimulation.. Parkinsonism Relat. Disord..

[70] Ory-Magne F., Corvol J.C., Azulay J.P., Bonnet A.M., Brefel-Courbon C., Damier P., Dellapina E., Destée A., Durif F., Galitzky M., Lebouvier T., Meissner W., Thalamas C., Tison F., Salis A., Sommet A., Viallet F., Vidailhet M., Rascol O. (2014). Withdrawing amantadine in dyskinetic patients with Parkinson disease: the AMANDYSK trial.. Neurology.

[71] Mizoguchi K., Yokoo H., Yoshida M., Tanaka T., Tanaka M. (1994). Amantadine increases the extracellular dopamine levels in the striatum by re-uptake inhibition and by N-methyl-D-aspartate antagonism.. Brain Res..

[72] Pahwa R., Tanner C.M., Hauser R.A., Sethi K., Isaacson S., Truong D., Struck L., Ruby A.E., McClure N.L., Went G.T., Stempien M.J. (2015). Amantadine extended release for levodopa-induced dyskinesia in Parkinson’s disease (EASED study).. Mov. Disord..

[73] Oertel W., Eggert K., Pahwa R., Tanner C.M., Hauser R.A., Trenkwalder C., Ehret R., Azulay J.P., Isaacson S., Felt L., Stempien M.J. (2017). Randomized, placebo-controlled trial of ADS-5102 (amantadine) extended-release capsules for levodopa-induced dyskinesia in Parkinson’s disease (EASE LID 3).. Mov. Disord..

[74] Pahwa R., Tanner C.M., Hauser R.A., Isaacson S.H., Nausieda P.A., Truong D.D., Agarwal P., Hull K.L., Lyons K.E., Johnson R., Stempien M.J. (2017). ADS-5102 (Amantadine) extended-release capsules for levodopa-induced dyskinesia in parkinson disease (EASE LID study): A randomized clinical trial.. JAMA Neurol..

[75] Mehta S.H., Pahwa R., Tanner C.M., Hauser R.A., Johnson R. (2021). Effects of gocovri (Amantadine) extended release capsules on non-motor symptoms in patients with Parkinson’s disease and dyskinesia.. Neurol. Ther..

[76] Müller T., Möhr J.D. (2019). Recent clinical advances in pharmacotherapy for levodopa-induced dyskinesia.. Drugs.

[77] Hauser R.A., Pahwa R., Wargin W.A., Souza-Prien C.J., McClure N., Johnson R., Nguyen J.T., Patni R., Went G.T. (2019). Pharmacokinetics of ADS-5102 (Amantadine) Extended release capsules administered once daily at bedtime for the treatment of dyskinesia.. Clin. Pharmacokinet..

[78] Malek N., Grosset D.G. (2015). Medication adherence in patients with Parkinson’s disease.. CNS Drugs.

[79] Perez-Lloret S., Rascol O. (2018). Efficacy and safety of amantadine for the treatment of L-DOPA-induced dyskinesia.. J. Neural Transm. (Vienna).

[80] Moreau C., Delval A., Tiffreau V., Defebvre L., Dujardin K., Duhamel A., Petyt G., Hossein-Foucher C., Blum D., Sablonnière B., Schraen S., Allorge D., Destée A., Bordet R., Devos D. (2013). Memantine for axial signs in Parkinson’s disease: A randomised, double-blind, placebo-controlled pilot study.. J. Neurol. Neurosurg. Psychiatry.

[81] Wictorin K., Widner H. (2016). Memantine and reduced time with dyskinesia in Parkinson’s Disease.. Acta Neurol. Scand..

[82] Wesnes K.A., Aarsland D., Ballard C., Londos E. (2015). Memantine improves attention and episodic memory in Parkinson’s disease dementia and dementia with Lewy bodies.. Int. J. Geriatr. Psychiatry.

[83] Fox S.H., Metman L.V., Nutt J.G., Brodsky M., Factor S.A., Lang A.E., Pope L.E., Knowles N., Siffert J. (2017). Trial of dextromethorphan/quinidine to treat levodopa-induced dyskinesia in Parkinson’s disease.. Mov. Disord..

[84] Silverdale M.A., Nicholson S.L., Crossman A.R., Brotchie J.M. (2005). Topiramate reduces levodopa-induced dyskinesia in the MPTP-lesioned marmoset model of Parkinson’s disease.. Mov. Disord..

[85] Kobylecki C., Hill M.P., Crossman A.R., Ravenscroft P. (2011). Synergistic antidyskinetic effects of topiramate and amantadine in animal models of Parkinson’s disease.. Mov. Disord..

[86] Kobylecki C., Burn D.J., Kass-Iliyya L., Kellett M.W., Crossman A.R., Silverdale M.A. (2014). Randomized clinical trial of topiramate for levodopa-induced dyskinesia in Parkinson’s disease.. Parkinsonism Relat. Disord..

[87] Goetz C.G., Stebbins G.T., Chung K.A., Nicholas A.P., Hauser R.A., Merkitch D., Stacy M.A. (2017). Topiramate as an adjunct to amantadine in the treatment of dyskinesia in parkinson’s disease: A randomized, double-blind, placebo-controlled multicenter study.. Mov. Disord..

[88] Lees A., Fahn S., Eggert K.M., Jankovic J., Lang A., Micheli F., Mouradian M.M., Oertel W.H., Olanow C.W., Poewe W., Rascol O., Tolosa E., Squillacote D., Kumar D. (2012). Perampanel, an AMPA antagonist, found to have no benefit in reducing “off” time in Parkinson’s disease.. Mov. Disord..

[89] Parkinson Study Group (2001). Evaluation of dyskinesias in a pilot, randomized, placebo-controlled trial of remacemide in advanced Parkinson disease.. Arch. Neurol..

[90] Iderberg H., Maslava N., Thompson A.D., Bubser M., Niswender C.M., Hopkins C.R., Lindsley C.W., Conn P.J., Jones C.K., Cenci M.A. (2015). Pharmacological stimulation of metabotropic glutamate receptor type 4 in a rat model of Parkinson’s disease and L-DOPA-induced dyskinesia: Comparison between a positive allosteric modulator and an orthosteric agonist.. Neuropharmacology.

[91] Charvin D., Di Paolo T., Bezard E., Gregoire L., Takano A., Duvey G., Pioli E., Halldin C., Medori R., Conquet F. (2018). An mGlu4-Positive Allosteric Modulator Alleviates Parkinsonism in Primates.. Mov. Disord..

[92] Pisani A., Bonsi P., Centonze D., Gubellini P., Bernardi G., Calabresi P. (2003). Targeting striatal cholinergic interneurons in Parkinson’s disease: focus on metabotropic glutamate receptors.. Neuropharmacology.

[93] Pourmirbabaei S., Dolatshahi M., Rahmani F. (2019). Pathophysiological clues to therapeutic applications of glutamate mGlu5 receptor antagonists in levodopa-induced dyskinesia.. Eur. J. Pharmacol..

[94] Berg D., Godau J., Trenkwalder C., Eggert K., Csoti I., Storch A., Huber H., Morelli-Canelo M., Stamelou M., Ries V., Wolz M., Schneider C., Di Paolo T., Gasparini F., Hariry S., Vandemeulebroecke M., Abi-Saab W., Cooke K., Johns D., Gomez-Mancilla B. (2011). AFQ056 treatment of levodopa-induced dyskinesias: results of 2 randomized controlled trials.. Mov. Disord..

[95] Stocchi F., Rascol O., Destee A., Hattori N., Hauser R.A., Lang A.E., Poewe W., Stacy M., Tolosa E., Gao H., Nagel J., Merschhemke M., Graf A., Kenney C., Trenkwalder C. (2013). AFQ056 in Parkinson patients with levodopa-induced dyskinesia: 13-week, randomized, dose-finding study.. Mov. Disord..

[96] Kumar R., Hauser R.A., Mostillo J., Dronamraju N., Graf A., Merschhemke M., Kenney C. (2016). Mavoglurant (AFQ056) in combination with increased levodopa dosages in Parkinson’s disease patients.. Int. J. Neurosci..

[97] Trenkwalder C., Stocchi F., Poewe W., Dronamraju N., Kenney C., Shah A., von Raison F., Graf A. (2016). Mavoglurant in Parkinson’s patients with l-Dopa-induced dyskinesias: Two randomized phase 2 studies.. Mov. Disord..

[98] Negida A., Ghaith H.S., Fala S.Y., Ahmed H., Bahbah E.I., Ebada M.A., Aziz M.A.E. (2021). Mavoglurant (AFQ056) for the treatment of levodopa-induced dyskinesia in patients with Parkinson’s disease: a meta-analysis.. Neurol. Sci..

[99] Bezard E., Pioli E.Y., Li Q., Girard F., Mutel V., Keywood C., Tison F., Rascol O., Poli S.M. (2014). The mGluR5 negative allosteric modulator dipraglurant reduces dyskinesia in the MPTP macaque model.. Mov. Disord..

[100] Rascol O., Ferreira J., Nègre-Pages L., Perez-Lloret S., Lacomblez L., Galitzky M., Lemarié J.C., Corvol J.C., Brotchie J.M., Bossi L. (2012). A proof-of-concept, randomized, placebo-controlled, multiple cross-overs (n-of-1) study of naftazone in Parkinson’s disease.. Fundam. Clin. Pharmacol..

[101] Corvol J.C., Durif F., Meissner W.G., Azulay J.P., Haddad R., Guimarães-Costa R., Mariani L.L., Cormier-Dequaire F., Thalamas C., Galitzky M., Boraud T., Debilly B., Eusebio A., Houot M., Dellapina E., Chaigneau V., Salis A., Lacomblez L., Benel L., Rascol O. (2019). Naftazone in advanced Parkinson’s disease: An acute L-DOPA challenge randomized controlled trial.. Parkinsonism Relat. Disord..

[102] Nicholson S.L., Brotchie J.M. (2002). 5-hydroxytryptamine (5-HT, serotonin) and Parkinson’s disease - opportunities for novel therapeutics to reduce the problems of levodopa therapy.. Eur. J. Neurol..

[103] Muñoz A., Li Q., Gardoni F., Marcello E., Qin C., Carlsson T., Kirik D., Di Luca M., Björklund A., Bezard E., Carta M. (2008). Combined 5-HT1A and 5-HT1B receptor agonists for the treatment of L-DOPA-induced dyskinesia.. Brain.

[104] Fox S.H. (2013). Non-dopaminergic treatments for motor control in Parkinson’s disease.. Drugs.

[105] Schneider R.B., Auinger P., Tarolli C.G., Iourinets J., Gil-Díaz M.C., Richard I.H. (2020). A trial of buspirone for anxiety in Parkinson’s disease: Safety and tolerability.. Parkinsonism Relat. Disord..

[106] Goetz C.G., Damier P., Hicking C., Laska E., Müller T., Olanow C.W., Rascol O., Russ H. (2007). Sarizotan as a treatment for dyskinesias in Parkinson’s disease: a double-blind placebo-controlled trial.. Mov. Disord..

[107] Goetz C.G., Laska E., Hicking C., Damier P., Müller T., Nutt J., Warren Olanow C., Rascol O., Russ H. (2008). Placebo influences on dyskinesia in Parkinson’s disease.. Mov. Disord..

[108] Bezard E., Tronci E., Pioli E.Y., Li Q., Porras G., Björklund A., Carta M. (2013). Study of the antidyskinetic effect of eltoprazine in animal models of levodopa-induced dyskinesia.. Mov. Disord..

[109] Svenningsson P., Rosenblad C., Af Edholm Arvidsson K., Wictorin K., Keywood C., Shankar B., Lowe D.A., Björklund A., Widner H. (2015). Eltoprazine counteracts l-DOPA-induced dyskinesias in Parkinson’s disease: a dose-finding study.. Brain.

[110] McFarthing K., Prakash N., Simuni T. (2019). CLINICAL TRIAL HIGHLIGHTS - DYSKINESIA.. J. Parkinsons Dis..

[111] Fisher R., Hikima A., Morris R., Jackson M.J., Rose S., Varney M.A., Depoortere R., Newman-Tancredi A. (2020). The selective 5-HT1A receptor agonist, NLX-112, exerts anti-dyskinetic and anti-parkinsonian-like effects in MPTP-treated marmosets.. Neuropharmacology.

[112] Depoortere R., Johnston T.H., Fox S.H., Brotchie J.M., Newman-Tancredi A. (2020). The selective 5-HT1A receptor agonist, NLX-112, exerts anti-dyskinetic effects in MPTP-treated macaques.. Parkinsonism Relat. Disord..

[113] Huot P., Johnston T.H., Fox S.H., Newman-Tancredi A., Brotchie J.M. (2015). The highly-selective 5-HT(1A) agonist F15599 reduces L-DOPA-induced dyskinesia without compromising anti-parkinsonian benefits in the MPTP-lesioned macaque.. Neuropharmacology.

[114] Leucht S., Corves C., Arbter D., Engel R.R., Li C., Davis J.M. (2009). Second-generation versus first-generation antipsychotic drugs for schizophrenia: a meta-analysis.. Lancet.

[115] Chang A., Fox S.H. (2016). Psychosis in Parkinson’s disease: Epidemiology, pathophysiology, and management.. Drugs.

[116] Brown T.M. (1999). Clozapine for drug-induced psychosis in Parkinson’s disease.. N. Engl. J. Med..

[117] Friedman J., Lannon M., Comella C., Factor S., Kurlan R., Richard I., Parsa M., Pfeiffer R., Davies R., Janko K., Brown D., Gardner I., Pearson N., Large K., Rast S., Oakes D., Goetz C., Paulson G., Marshall F., Greenamyre J. (1999). Low-dose clozapine for the treatment of drug-induced psychosis in Parkinson’s disease.. N. Engl. J. Med..

[118] Yaw T.K., Fox S.H., Lang A.E. (2015). Clozapine in Parkinsonian rest tremor: A review of outcomes, adverse reactions, and possible mechanisms of action.. Mov. Disord. Clin. Pract. (Hoboken).

[119] Bonuccelli U., Ceravolo R., Salvetti S., D’Avino C., Del Dotto P., Rossi G., Murri L. (1997). Clozapine in Parkinson’s disease tremor. Effects of acute and chronic administration.. Neurology.

[120] Durif F., Debilly B., Galitzky M., Morand D., Viallet F., Borg M., Thobois S., Broussolle E., Rascol O. (2004). Clozapine improves dyskinesias in Parkinson disease: a double-blind, placebo-controlled study.. Neurology.

[121] Honigfeld G., Arellano F., Sethi J., Bianchini A., Schein J. (1998). Reducing clozapine-related morbidity and mortality: 5 years of experience with the Clozaril National Registry.. J. Clin. Psychiatry.

[122] Devinsky O., Honigfeld G., Patin J. (1991). Clozapine-related seizures.. Neurology.

[123] Meltzer H.Y., Mills R., Revell S., Williams H., Johnson A., Bahr D., Friedman J.H. (2010). Pimavanserin, a serotonin(2A) receptor inverse agonist, for the treatment of parkinson’s disease psychosis.. Neuropsychopharmacology.

[124] Cummings J., Isaacson S., Mills R., Williams H., Chi-Burris K., Corbett A., Dhall R., Ballard C. (2014). Pimavanserin for patients with Parkinson’s disease psychosis: a randomised, placebo-controlled phase 3 trial.. Lancet.

[125] Espay A.J., Guskey M.T., Norton J.C., Coate B., Vizcarra J.A., Ballard C., Factor S.A., Friedman J.H., Lang A.E., Larsen N.J., Andersson C., Fredericks D., Weintraub D. (2018). Pimavanserin for Parkinson’s Disease psychosis: Effects stratified by baseline cognition and use of cognitive-enhancing medications.. Mov. Disord..

[126] Chendo I., Ferreira J.J. (2016). Pimavanserin for the treatment of Parkinson’s disease psychosis.. Expert Opin. Pharmacother..

[127] Markham A. (2016). Pimavanserin: First Global Approval.. Drugs.

[128] Braak H., Del Tredici K. (2017). Neuropathological Staging of Brain Pathology in Sporadic Parkinson’s disease: Separating the Wheat from the Chaff.. J. Parkinsons Dis..

[129] Karachi C., Grabli D., Bernard F.A., Tandé D., Wattiez N., Belaid H., Bardinet E., Prigent A., Nothacker H.P., Hunot S., Hartmann A., Lehéricy S., Hirsch E.C., François C. (2010). Cholinergic mesencephalic neurons are involved in gait and postural disorders in Parkinson disease.. J. Clin. Invest..

[130] Yarnall A., Rochester L., Burn D.J. (2011). The interplay of cholinergic function, attention, and falls in Parkinson’s disease.. Mov. Disord..

[131] Henderson E.J., Lord S.R., Brodie M.A., Gaunt D.M., Lawrence A.D., Close J.C., Whone A.L., Ben-Shlomo Y. (2016). Rivastigmine for gait stability in patients with Parkinson’s disease (ReSPonD): a randomised, double-blind, placebo-controlled, phase 2 trial.. Lancet Neurol..

[132] Li Z., Yu Z., Zhang J., Wang J., Sun C., Wang P., Zhang J. (2015). Impact of rivastigmine on cognitive dysfunction and falling in Parkinson’s disease patients.. Eur. Neurol..

[133] McDonald J., Pourcher E., Nadeau A., Corbeil P. (2018). A Randomized trial of oral and transdermal rivastigmine for postural instability in Parkinson disease dementia.. Clin. Neuropharmacol..

[134] van Mierlo T.J.M., Foncke E.M.J., Post B., Schmand B.A., Bloem B.R., van Harten B., Tissingh G., Munts A.G., de Haan R.J., de Bie R.M.A. (2021). Rivastigmine for minor visual hallucinations in Parkinson’s disease: A randomized controlled trial with 24 months follow-up.. Brain Behav..

[135] Espay A.J., Marsili L., Mahajan A., Sturchio A., Pathan R., Pilotto A., Elango D.S., Pezous N., Masellis M., Gomez-Mancilla B. (2021). Rivastigmine in Parkinson’s disease dementia with orthostatic hypotension.. Ann. Neurol..

[136] Dubois B., Tolosa E., Katzenschlager R., Emre M., Lees A.J., Schumann G., Pourcher E., Gray J., Thomas G., Swartz J., Hsu T., Moline M.L. (2012). Donepezil in Parkinson’s disease dementia: a randomized, double-blind efficacy and safety study.. Mov. Disord..

[137] Chung K.A., Lobb B.M., Nutt J.G., Horak F.B. (2010). Effects of a central cholinesterase inhibitor on reducing falls in Parkinson disease.. Neurology.

[138] Mancini M., Chung K., Zajack A., Martini D.N., Ramsey K., Lapidus J., Horak F.B., Nutt J.G. (2019). Effects of augmenting cholinergic neurotransmission on balance in Parkinson’s disease.. Parkinsonism Relat. Disord..

[139] Singer W., Opfer-Gehrking T.L., McPhee B.R., Hilz M.J., Bharucha A.E., Low P.A. (2003). Acetylcholinesterase inhibition: a novel approach in the treatment of neurogenic orthostatic hypotension.. J. Neurol. Neurosurg. Psychiatry.

[140] Schreglmann S.R., Büchele F., Sommerauer M., Epprecht L., Kägi G., Hägele-Link S., Götze O., Zimmerli L., Waldvogel D., Baumann C.R. (2017). Pyridostigmine bromide versus fludrocortisone in the treatment of orthostatic hypotension in Parkinson’s disease - a randomized controlled trial.. Eur. J. Neurol..

[141] Villafane G., Cesaro P., Rialland A., Baloul S., Azimi S., Bourdet C., Le Houezec J., Macquin-Mavier I., Maison P. (2007). Chronic high dose transdermal nicotine in Parkinson’s disease: an open trial.. Eur. J. Neurol..

[142] Villafane G., Thiriez C., Audureau E., Straczek C., Kerschen P., Cormier-Dequaire F., Van Der Gucht A., Gurruchaga J.M., Quéré-Carne M., Evangelista E., Paul M., Defer G., Damier P., Remy P., Itti E., Fénelon G. (2018). High-dose transdermal nicotine in Parkinson’s disease patients: a randomized, open-label, blinded-endpoint evaluation phase 2 study.. Eur. J. Neurol..

[143] Lieberman A., Lockhart T.E., Olson M.C., Smith Hussain V.A., Frames C.W., Sadreddin A., McCauley M., Ludington E. (2019). Nicotine bitartrate reduces falls and freezing of gait in Parkinson disease: A reanalysis.. Front. Neurol..

[144] Di Paolo T., Grégoire L., Feuerbach D., Elbast W., Weiss M., Gomez-Mancilla B. (2014). AQW051, a novel and selective nicotinic acetylcholine receptor α7 partial agonist, reduces l-Dopa-induced dyskinesias and extends the duration of l-Dopa effects in parkinsonian monkeys.. Parkinsonism Relat. Disord..

[145] Trenkwalder C., Berg D., Rascol O., Eggert K., Ceballos-Baumann A., Corvol J.C., Storch A., Zhang L., Azulay J.P., Broussolle E., Defebvre L., Geny C., Gostkowski M., Stocchi F., Tranchant C., Derkinderen P., Durif F., Espay A.J., Feigin A., Houeto J.L., Schwarz J., Di Paolo T., Feuerbach D., Hockey H.U., Jaeger J., Jakab A., Johns D., Linazasoro G., Maruff P., Rozenberg I., Sovago J., Weiss M., Gomez-Mancilla B. (2016). A placebo-controlled trial of AQW051 in patients with moderate to severe levodopa-induced dyskinesia.. Mov. Disord..

[146] Arbouw M.E., Movig K.L., Koopmann M., Poels P.J., Guchelaar H.J., Egberts T.C., Neef C., van Vugt J.P. (2010). Glycopyrrolate for sialorrhea in Parkinson disease: a randomized, double-blind, crossover trial.. Neurology.

[147] Mestre T.A., Freitas E., Basndwah A., Lopez M.R., de Oliveira L.M., Al-Shorafat D.M., Zhang T., Lui J.P., Grimes D., Fox S.H. (2020). Glycopyrrolate improves disability from sialorrhea in Parkinson’s disease: A 12-week controlled trial.. Mov. Disord..

[148] Lloret S.P., Nano G., Carrosella A., Gamzu E., Merello M. (2011). A double-blind, placebo-controlled, randomized, crossover pilot study of the safety and efficacy of multiple doses of intra-oral tropicamide films for the short-term relief of sialorrhea symptoms in Parkinson’s disease patients.. J. Neurol. Sci..

[149] Mills R., Bahroo L., Pagan F. (2015). An update on the use of botulinum toxin therapy in Parkinson’s disease.. Curr. Neurol. Neurosci. Rep..

[150] Rahimi F., Samotus O., Lee J., Jog M. (2015). Effective management of upper limb parkinsonian tremor by incobotulinumtoxin A injections using sensor-based biomechanical patterns.. Tremor Other Hyperkinet. Mov. (N. Y.).

[151] Mittal S.O., Machado D., Richardson D., Dubey D., Jabbari B. (2017). Botulinum toxin in Parkinson disease tremor: A randomized, double-blind, placebo-controlled study with a customized injection approach.. Mayo Clin. Proc..

[152] Bruno V., Freitas M.E., Mancini D., Lui J.P., Miyasaki J., Fox S.H. (2018). Botulinum toxin type A for pain in advanced Parkinson’s disease.. Can. J. Neurol. Sci..

[153] Knüpfer S.C., Schneider S.A., Averhoff M.M., Naumann C.M., Deuschl G., Jünemann K.P., Hamann M.F. (2016). Preserved micturition after intradetrusor onabotulinumtoxin A injection for treatment of neurogenic bladder dysfunction in Parkinson’s disease.. BMC Urol..

[154] Vurture G., Peyronnet B., Feigin A., Biagioni M.C., Gilbert R., Rosenblum N., Frucht S., Di Rocco A., Nitti V.W., Brucker B.M. (2018). Outcomes of intradetrusor onabotulinum toxin A injection in patients with Parkinson’s disease.. Neurourol. Urodyn..

[155] Egevad G., Petkova V.Y., Vilholm O.J. (2014). Sialorrhea in patients with Parkinson’s disease: safety and administration of botulinum neurotoxin.. J. Parkinsons Dis..

[156] Srivanitchapoom P., Pandey S., Hallett M. (2014). Drooling in Parkinson’s disease: a review.. Parkinsonism Relat. Disord..

[157] Petracca M., Guidubaldi A., Ricciardi L., Ialongo T., Del Grande A., Mulas D., Di Stasio E., Bentivoglio A.R. (2015). Botulinum Toxin A and B in sialorrhea: Long-term data and literature overview.. Toxicon..

[158] Jost W.H., Friedman A., Michel O., Oehlwein C., Slawek J., Bogucki A., Ochudlo S., Banach M., Pagan F., Flatau-Baqué B., Csikós J., Cairney C.J., Blitzer A. (2019). SIAXI: Placebo-controlled, randomized, double-blind study of incobotulinumtoxinA for sialorrhea.. Neurology.

[159] Jost W.H., Friedman A., Michel O., Oehlwein C., Slawek J., Bogucki A., Ochudlo S., Banach M., Pagan F., Flatau-Baqué B., Dorsch U., Csikós J., Blitzer A. (2020). Long-term incobotulinumtoxinA treatment for chronic sialorrhea: Efficacy and safety over 64 weeks.. Parkinsonism Relat. Disord..

[160] Isaacson S.H., Ondo W., Jackson C.E., Trosch R.M., Molho E., Pagan F., Lew M., Dashtipour K., Clinch T., Espay A.J. (2020). Safety and efficacy of rimabotulinumtoxinb for treatment of sialorrhea in adults: A randomized clinical Trial.. JAMA Neurol..

[161] Lewitt P.A. (2012). Norepinephrine: the next therapeutics frontier for Parkinson’s disease.. Transl. Neurodegener..

[162] Espay A.J., LeWitt P.A., Kaufmann H. (2014). Norepinephrine deficiency in Parkinson’s disease: the case for noradrenergic enhancement.. Mov. Disord..

[163] Hauser R.A., Hewitt L.A., Isaacson S. (2014). Droxidopa in patients with neurogenic orthostatic hypotension associated with Parkinson’s disease (NOH306A).. J. Parkinsons Dis..

[164] Hauser R.A., Isaacson S., Lisk J.P., Hewitt L.A., Rowse G. (2015). Droxidopa for the short-term treatment of symptomatic neurogenic orthostatic hypotension in Parkinson’s disease (nOH306B).. Mov. Disord..

[165] Hauser R.A., Heritier S., Rowse G.J., Hewitt L.A., Isaacson S.H. (2016). Droxidopa and reduced falls in a trial of Parkinson disease patients with neurogenic orthostatic hypotension.. Clin. Neuropharmacol..

[166] François C., Hauser R.A., Aballéa S., Dorey J., Kharitonova E., Hewitt L.A. (2016). Cost-effectiveness of droxidopa in patients with neurogenic orthostatic hypotension: post-hoc economic analysis of Phase 3 clinical trial data.. J. Med. Econ..

[167] Zhao S., Cheng R., Zheng J., Li Q., Wang J., Fan W., Zhang L., Zhang Y., Li H., Liu S. (2015). A randomized, double-blind, controlled trial of add-on therapy in moderate-to-severe Parkinson’s disease.. Parkinsonism Relat. Disord..

[168] Ostock C.Y., Hallmark J., Palumbo N., Bhide N., Conti M., George J.A., Bishop C. (2015). Modulation of L-DOPA’s antiparkinsonian and dyskinetic effects by α2-noradrenergic receptors within the locus coeruleus.. Neuropharmacology.

[169] Espay A.J., Dwivedi A.K., Payne M., Gaines L., Vaughan J.E., Maddux B.N., Slevin J.T., Gartner M., Sahay A., Revilla F.J., Duker A.P., Shukla R. (2011). Methylphenidate for gait impairment in Parkinson disease: a randomized clinical trial.. Neurology.

[170] Moreau C., Delval A., Defebvre L., Dujardin K., Duhamel A., Petyt G., Vuillaume I., Corvol J.C., Brefel-Courbon C., Ory-Magne F., Guehl D., Eusebio A., Fraix V., Saulnier P.J., Lagha-Boukbiza O., Durif F., Faighel M., Giordana C., Drapier S., Maltête D., Tranchant C., Houeto J.L., Debû B., Sablonniere B., Azulay J.P., Tison F., Rascol O., Vidailhet M., Destée A., Bloem B.R., Bordet R., Devos D. (2012). Methylphenidate for gait hypokinesia and freezing in patients with Parkinson’s disease undergoing subthalamic stimulation: a multicentre, parallel, randomised, placebo-controlled trial.. Lancet Neurol..

[171] Delval A., Moreau C., Bleuse S., Guehl D., Bestaven E., Guillaud E., Dujardin K., Defebvre L., Devos D. (2015). Gait and attentional performance in freezers under methylphenidate.. Gait Posture.

[172] Prokic E.J., Stanford I.M., Woodhall G.L., Williams A.C., Hall S.D. (2019). Bradykinesia is driven by cumulative beta power during continuous movement and alleviated by gabaergic modulation in Parkinson’s disease.. Front. Neurol..

[173] Siriwardena A.N., Apekey T., Tilling M., Dyas J.V., Middleton H., Ørner R. (2010). General practitioners’ preferences for managing insomnia and opportunities for reducing hypnotic prescribing.. J. Eval. Clin. Pract..

[174] Chen Y.Y., Sy H.N., Wu S.L. (2008). Zolpidem improves akinesia, dystonia and dyskinesia in advanced Parkinson’s disease.. J. Clin. Neurosci..

[175] Huang H.Y., Hsu Y.T., Wu Y.C., Chiou S.M., Kao C.H., Tsai M.C., Tsai C.H. (2012). Zolpidem improves neuropsychiatric symptoms and motor dysfunction in a patient with Parkinson’s disease after deep brain stimulation.. Acta Neurol. Taiwan..

[176] Evidente V.G. (2002). Zolpidem improves dystonia in “Lubag” or X-linked dystonia-parkinsonism syndrome.. Neurology.

[177] Bullock A., Kaul I., Li S., Silber C., Doherty J., Kanes S.J. (2021). Zuranolone as an oral adjunct to treatment of Parkinsonian tremor: A phase 2, open-label study.. J. Neurol. Sci..

[178] Benarroch E. (2007). Endocannabinoids in basal ganglia circuits: implications for Parkinson disease.. Neurology.

[179] McSherry J.W., Carroll C.B., Zajicek J., Teare L., Bain P. (2005). Cannabis for dyskinesia in Parkinson disease: a randomized double-blind crossover study.. Neurology.

[180] Sieradzan K.A., Fox S.H., Hill M., Dick J.P., Crossman A.R., Brotchie J.M. (2001). Cannabinoids reduce levodopa-induced dyskinesia in Parkinson’s disease: a pilot study.. Neurology.

[181] Koppel B.S., Brust J.C., Fife T., Bronstein J., Youssof S., Gronseth G., Gloss D. (2014). Systematic review: efficacy and safety of medical marijuana in selected neurologic disorders: report of the Guideline Development Subcommittee of the American Academy of Neurology.. Neurology.

[182] de Faria S.M., de Morais Fabrício D., Tumas V., Castro P.C., Ponti M.A., Hallak J.E., Zuardi A.W., Crippa J.A.S., Chagas M.H.N. (2020). Effects of acute cannabidiol administration on anxiety and tremors induced by a Simulated Public Speaking Test in patients with Parkinson’s disease.. J. Psychopharmacol..

[183] Prast H., Tran M.H., Fischer H., Kraus M., Lamberti C., Grass K., Philippu A. (1999). Histaminergic neurons modulate acetylcholine release in the ventral striatum: role of H3 histamine receptors.. Naunyn Schmiedebergs Arch. Pharmacol..

[184] Johnston T.H., van der Meij A., Brotchie J.M., Fox S.H. (2010). Effect of histamine H2 receptor antagonism on levodopa-induced dyskinesia in the MPTP-macaque model of Parkinson’s disease.. Mov. Disord..

[185] Mestre T.A., Shah B.B., Connolly B.S., de Aquino C., Al Dhakeel A., Walsh R., Ghate T., Lui J.P., Fox S.H. (2014). Famotidine, a Histamine H2 Receptor Antagonist, Does Not Reduce Levodopa-Induced Dyskinesia in Parkinson’s Disease: A Proof-of-Concept Study.. Mov. Disord. Clin. Pract. (Hoboken).

[186] Trenkwalder C., Chaudhuri K.R., Martinez-Martin P., Rascol O., Ehret R., Vališ M., Sátori M., Krygowska-Wajs A., Marti M.J., Reimer K., Oksche A., Lomax M., DeCesare J., Hopp M. (2015). Prolonged-release oxycodone-naloxone for treatment of severe pain in patients with Parkinson’s disease (PANDA): a double-blind, randomised, placebo-controlled trial.. Lancet Neurol..

[187] Madeo G., Schirinzi T., Natoli S., Pierantozzi M., Stefani A., Dauri M., Pisani A. (2015). Efficacy and safety profile of prolonged release oxycodone in combination with naloxone (OXN PR) in Parkinson’s disease patients with chronic pain.. J. Neurol..

[188] Papay K., Xie S.X., Stern M., Hurtig H., Siderowf A., Duda J.E., Minger J., Weintraub D. (2014). Naltrexone for impulse control disorders in Parkinson disease: a placebo-controlled study.. Neurology.

